# Association between statin use and risk of incident cancer in healthy older adults: a target trial emulation using data from a multicentre, randomised trial of community-dwelling older adults in Australia and the USA

**DOI:** 10.1016/j.eclinm.2025.103746

**Published:** 2026-01-12

**Authors:** Gebiso Roba Debele, Najmeh Davoodian, Mojtaba Lotfaliany, Rory Wolfe, Michael Berk, Andrew M. Tonkin, Peter Gibbs, Zhen Zhou, Robyn L. Woods, Suzanne G. Orchard, A.R.M Saifuddin Ekram, Anne M. Murray, Mark Nelson, Jeremy L. Millar, Aaron R. Kent, Wee Loon Ong, Christopher M. Reid, Raj C. Shah, Andrew Chan, Daniel Clayton-Chubb, Sophia Zoungas, John J. McNeil, Mohammadreza Mohebbi

**Affiliations:** a Deakin University, IMPACT-the Institute for Mental and Physical Health and Clinical Translation, School of Medicine, Geelong, Victoria, Australia; b Department of Public Health, College of Health Science, Mattu University, Mattu, Ethiopia; c Department of Psychiatry, Orygen, The National Centre of Excellence in Youth Health, and the Florey Institute for Neuroscience and Mental Health, University of Melbourne, Melbourne, Victoria, Australia; d School of Public Health and Preventive Medicine, Monash University, Melbourne, Victoria, Australia; e Department of Gastroenterology, Alfred Health, Melbourne, Australia; f Menzies Institute for Medical Research, University of Tasmania, Hobart, Tasmania, Australia; g Berman Center for Outcomes and Clinical Research, Hennepin Healthcare Research Institute, Hennepin Healthcare, Minneapolis, MN, USA; h Department of Family and Preventive Medicine and The Rush Alzheimer's Disease Center, Rush University Medical Center, Chicago, IL, USA; i Division of Gastroenterology, Massachusetts General Hospital and Harvard Medical School, Boston, MA, USA; j Personalised Oncology Division, Walter and Eliza Hall Institute, Melbourne, Victoria, Australia; k Biostatistics Unit, Faculty of Health, Deakin University, Geelong, Victoria, Australia; l Alfred Health Radiation Oncology, Alfred Hospital, Melbourne, Victoria, Australia

**Keywords:** Statin use, Cancer incidence, Health aging, Target trial emulation

## Abstract

**Background:**

The evidence on whether statin use affects cancer incidence is inconclusive and limited among apparently healthy older adults. This study emulated a target trial comparing statin initiators versus non-initiators, with further analyses stratified by statin lipophilicity.

**Methods:**

We conducted a target trial emulation using ASPREE (ASPirin in Reducing Events in the Elderly) and its extended observational data (ASPREE-XT). ASPREE was a placebo-controlled trial of low-dose aspirin in 19,114 older adults predominantly ≥70 years of age in Australia and the United States, who had no cardiovascular events, dementia, and independence-limiting physical disability at enrolment. We emulated a target trial of statin initiators versus non-initiators, following a prespecified target protocol. Key exclusion criteria were prior cardiovascular or cerebrovascular disease, high bleeding risk, conditions likely to limit 5-year survival, and anemia. The primary outcome was any-incident cancer and site-specific cancer, as adjudicated by an expert panel. Inverse probability weighting (IPW) was applied to adjust for predefined confounders including sociodemographic, clinical, and anthropometric factors, comorbidities, and co-medications to achieve balance between treatment groups.

**Findings:**

Participants were enrolled from March 01, 2010, to December 31, 2014, with follow-up to June 12, 2017, during the trial phase and then extended to January 08, 2022, as observational study. Of 12,557 eligible participants, 1596 (12.7%) initiated statin, including 882 (7.0% lipophilic and 714 (5.7%) hydrophilic statin. Over a median follow-up of 8.3 years (IQR; 6.5–9.5), the cumulative incidence of cancer per 1000 person-years was 16.0 (95% CI: [13.8–18.3]) in statin initiators and 21.6 (95% CI: [20.6–22.6]) in the non-initiators. Statin use was associated with a lower risk of cancer (Sub-distribution Hazard Ratio (SHR): 0.70 95% CI [0.59–0.82]), metastatic (SHR: 0.70 95% CI [0.52–0.93]) and non-metastatic (SHR: 0.71 95% CI [0.58–0.87]) cancers. This association remained significant for lipophilic statins (metastatic (SHR: 0.65 95% CI [0.45–0.94]) and non-metastatic (SHR: 0.64 95% CI [0.49–0.84]) cancers), but not for hydrophilic statins (metastatic (SHR: 0.77 95% CI [0.52–1.13]) and non-metastatic (SHR: 0.80 95% CI [0.61–1.06]) cancers). Among site-specific cancers, prostate cancer (SHR 0.67; 95% CI 0.47–0.95) and breast cancer (SHR 0.55; 95% CI 0.33–0.93) showed significant association. The estimated number needed to treat associated with statin use was 31 (95% CI: 25–47) for any cancer, 56 (95% CI: 44–87) metastatic cancer, and 72 (95% CI: 46–100) non-metastatic cancer over a median follow-up of 8.3 years.

**Interpretation:**

In this target trial emulation, statin use was associated with lower cancer incidence in older adults, with potential differences by cancer type and statin lipophilicity. These findings highlight the need for long-term randomised control trial to confirm this association. Potential for unmeasured confounding and bias due to the non-randomised observational design was a study limitation, and the inclusion of healthy participants may limit the generalizability of the findings.

**Funding:**

10.13039/100000049National Institute on Aging, 10.13039/100000054National Cancer Institute, 10.13039/501100000925National Health and Medical Research Council of Australia, 10.13039/501100001779Monash University, 10.13039/100008018Victorian Cancer Agency and Deakin University Postgraduate Research Scholarship.

**Research in context**

**Evidence before this study**

We searched PubMed, Cochrane Central, and Embase for articles published from inception to April 01/2025 and those specifically evaluating the effects or associations of statin regimens on cancer incidence. The search strategy included a combination of the following keywords: (statin OR hydroxymethylglutaryl coenzyme A reductase inhibitors) AND (cancer OR malignant neoplasm OR neoplasm) AND (aged OR older adults OR older people) AND (observational study OR randomized controlled trial-(RCT)). Several observational studies have explored the association between statin use and cancer risk in older adults. However, their findings have been inconsistent, at least in part due to limitations such as small sample sizes, short follow-up durations, and biases such as selection and confounding bias have further compounded these results. Notably, none of the studies have utilized adjudicated cancer outcomes, raising concerns about potential misclassification of cancer status. We also found no RCT to date that specifically investigated the effect of lipophilicity on statins chemo-preventive potential against cancer in older adults.

**Added value of this study**

In this study we emulated a target trial by using high-quality post-randomization data from the ASPirin in Reducing Events in the Elderly (ASPREE) trial of older adults in Australia and the USA. The ASPREE study was a double-blind, randomized, placebo-controlled trial of 19,114 older adults without cardiovascular events, dementia, or disability, which ran from March 2010 until 2017, after which it was extended as an observational cohort study through January 2022. Statin use was associated with a reduced risk of cancer including metastatic cancer, non-metastatic cancer and hormone prostate cancer and breast cancer. Moreover, while lipophilic statins were associated with a lower risk of metastatic and non-metastatic cancers, hydrophilic statins did not show this association.

Statin use was associated with a lower risk of cancer, including metastatic and non-metastatic disease, as well as hormone-sensitive malignancies such as prostate and breast cancer. Moreover, lipophilic statins were associated with a reduced cancer risk, whereas hydrophilic statins showed no such association. These observations emphasize the importance of pharmacological properties, especially tissue permeability, in contributing to statins’ role against cancer.

**Implications of all the available evidence**

When considered alongside the limited existing evidence, our findings support a potential role for statins in reducing the risk of cancer. Moreover, these associations may differ by statin lipophilicity and site-specific cancers. However, these results should be interpreted cautiously given the possibility of residual confounding, which limits causal inference.

## Introduction

Cancer remains a global public health burden, with nearly 20 million new cases and 10 million deaths in 2022.^1^ Older adults (≥60 years of age) account for 64% of cases and over 71% of cancer-related deaths.^2^ As cancer rates increase with age, effective preventive strategies can contribute to healthy aging.^3^ Evidence from large randomised controlled trial (RCT) supports the use of statins to prevent cardiovascular disease (CVD) events and reduce all-cause mortality.^4^ However, there is inconsistent evidence regarding their long-term effect on cancer prevention. Statins, primarily recognised for their cholesterol-lowering ability through the inhibition of the 3-hydroxy-3-methylglutaryl-coenzyme A (HMG-CoA) reductase enzyme, are a potential adjunctive therapy for the prevention and treatment of several off target disorders including cancer.^5^^,^^6^ Statins have a range of pleiotropic actions, including anti-inflammatory, antioxidant, anti-proliferative, and immunomodulatory effects, which may confer clinically significant anticancer benefits.^7^ Statins differ in solubility; a water soluble hydrophilic statins are liver-selective, while lipophilic statins (fat soluble) have greater tissue permeability, potentially enhancing their off-target effects.^8^

Despite the reported potential of statins as anticancer agents, the current literature remains inconclusive. Some studies have suggested preventive benefits,^9^, ^10^, ^11^, ^12^ although others found no association.^13^ The potential difference may be attributed to differences in the study population, design and biases inherent in observational research. These include (i) confounding or indication bias, arising from incomplete adjustment for factors related to statin initiation (ii) selection bias, from including statin initiators who may already have a cancer diagnosis,^14^ and (iii) younger cohorts outside of the window of peak risk. Furthermore, the above studies lacked adjudication of cancer outcomes and had short follow-up periods that averaged less than five years.

Therefore, a thorough assessment of the association between statin and the incidence of cancer in older adults would be valuable for both clinical and public health perspectives. In the absence of large-scale RCT in this population, well-designed observational studies that emulate target trial could guide decision-making and public health policy. This approach fosters interpretation by explicitly defining eligibility criteria, treatment regimens, and follow-up protocols.^15^ Thus, we emulated a target trial comparing statin initiators and non-initiators using data from ASPirin in Reducing Events in the Elderly (ASPREE) trial and its extended observational follow-up (ASPREE-XT), with rigorous ascertainment and adjudication of cancer events. The primary objective of this study was to investigate the association of statin use and incident cancer in community-dwelling older adults, with a secondary analysis evaluating whether these effects differ based on the lipophilicity of the statins.

## Methods

### Study design and participants

We employed a target trial emulation framework^15^ to replicate the design and analytical principles of a RCT comparing statin initiation with non-initiation, based on a prespecified protocol (Table S1). We used data from the ASPREE study, an RCT of daily 100 mg aspirin in 19,114 adults aged ≥70 years in Australia and the USA (≥65 years) without prior CVD events, dementia, or independence-limiting physical disability, that was conducted from March 2010 to June 2017, with extended observational follow-up post-trial until January 2022 (the ASPREE-XT study).^16^^,^^17^ Participants with a prior history of cancer, reported in 19% of those enrolled in ASPREE, were not excluded, provided they were in good health, free of major diseases, and had a life expectancy of at least five years.^18^ The design and exclusion criteria of the ASPREE study have been described in detail in previous publications.^16^^,^^19^ The prespecified primary endpoint of the trial studied the effect of daily low-dose aspirin on disability-free survival.^16^ The use of statins, including specific types of statins, was recorded as a concurrent prescription medication at enrolment and each annual visit.

### Exposure, outcome definitions, and confounder selection

Statin therapy was defined as treatment with any dose of atorvastatin, fluvastatin, lovastatin, pitavastatin, simvastatin, pravastatin and rosuvastatin. Statin use was verified by participants bringing to visits their prescribed medications or a list of them, and where possible, by review of the participant's medical record at their primary care clinic. For the target trial, prevalent statin users at randomisation were excluded and new statin initiators during the trial period identified before events with the time zero for analysis set to the first annual visit and followed until January 2022.^20^ This design ensured a clear temporal alignment with statin initiation preceding outcome assessment. The primary outcome was any-incident cancer and site-specific cancer determined by the ASPREE cancer endpoint adjudication committee.^18^ Details of the cancer endpoint definition and adjudication criteria can be found in Table S2. A new cancer event was one that could be localized or metastatic at presentation or a metastatic recurrence of a pre-existing non-metastatic cancer. If the cancer type differed (different primary sites or different pathology), each participant could contribute two or more unique cancer events. For instance, a participant diagnosed with breast cancer post-randomisation who later developed colon cancer was considered to have two cancers. If participants had more than one form of anatomical cancer (e.g., lobular carcinoma versus ductal carcinoma of the breast), just the initial report of each type of cancer was included in the analysis of cancer of any type (overall cancer). If death was attributed to cancer, the malignancy most likely responsible was identified. In participants with a prior cancer history, new cancer types were classified as incident cancer, whereas local recurrences of the same type were not. Based on expert knowledge and previous study,^10^ sociodemographic, clinical and anthropometric factors, comorbidities and chronic conditions and relevant medications were used as confounders for propensity score calculation and the causal relationships between the included covariates, statin and outcome were summarised in Figure S1. Missing covariate data (up to 2.5%) was imputed using single predictive means matching ().

### Target trail emulation (TTE)

While RCTs remain the gold standard for establishing causal relationships, well-emulated observational studies can offer valuable insights to inform clinical and public health decision-making.^21^ TTE applies RCT principles to observational data analysis by aligning study design and statistical methods with a hypothetical trial, while adjusting for non-randomisation through measured confounders. It involves a two-step approach that begins with developing a protocol for a hypothetical RCT that include specifying eligibility criteria, treatment strategies and assignment, follow-up, outcomes, causal contrasts, and analysis plans.^15^ The second step is replicating these elements using observational data by identifying individuals who meet the eligibility criteria, assigning them to treatment groups, and following them from assignment to the occurrence of an outcome of interest or end of follow-up. Accordingly, we emulated a target trial comparing statin initiators to non-initiators and reported the study following the TARGET guideline.^22^

### Statistical analysis

We determined the detectable effect size at 80% power assuming 0.05 alpha, using Freedman's approach for power calculation.^23^ The sample size can detect a minimum of 18%, 25%, and 30% reduction for any cancer, non-metastatic cancer, and metastatic cancer, respectively (Table S5). This indicates that our sample size is sufficient to detect the observed moderate effects with 80% power. Two analytical approaches were employed in this study: intention-to-treat (ITT) analysis using time fixed statin exposure according to initiation status and a time dependent (TD) exposure analysis that accounted for different time of statin initiation, subsequent discontinuation and re-initiation. The ITT analysis ignores changes in treatment over time and thus estimates the effects of initiation of an intended course of statin treatment. Conversely, the TD analysis aims to estimate the cumulative associated effects of ongoing statin following initiation and can mitigate immortal time bias by aligning the exposure status with the actual timing of treatment.^24^ We computed stabilized inverse probability of treatment weights (IPTW) using propensity scores for statin prescription based on confounders to replicate randomisation. To reduce the impact of extreme weights on the estimate and variance, we truncated weights at the upper and lower 0.5 percentiles. We evaluated the covariate balance between treatment groups by using the standardized differences of attributes before and after weighting, with values under 0.1 indicating good balance.^25^

Both ITT and TD analysis were fitted using the marginal Fine–Gray sub-distribution hazard model with non-cancer death as a competing risk to estimate sub-distribution hazard ratios (SHRs). The proportional hazard assumption was evaluated through time-varying coefficient plots and log–log plot (Figure S2). The 95% confidence intervals (CIs) for the SHRs were computed using a robust (sandwich) variance estimator. All comparisons are two-tailed, with P < 0.05 considered statistically significant. In a secondary analysis, the three groups: lipophilic statins, hydrophilic statins, and non-statin initiators were compared to determine if effects differed based on the lipophilicity status of the statin. The analysis followed the same steps as for the primary analysis, except that logistic regression was extended to multinomial logistic regression to compute the propensity score. Site-specific cancer and statin lipophilicity subgroup analyses were adjusted for multiple comparisons using the Benjamini–Hochberg procedure, controlling the false discovery rate at < 0.05. The number needed to treat (NNT) was calculated as the inverse of the absolute risk reduction. All statistical analyses were conducted using R software, version 4.3.2 (R Foundation for Statistical Computing, Vienna, Austria).

### Subgroup and sensitivity analysis

The association between statin use and cancer was estimated across subgroups defined by age, sex, race or ethnicity, family history of cancer, personal history of cancer, smoking status, alcohol consumption, body mass index (BMI) categories, diabetes mellitus (DM), hypertension (HTN) CVD risk,^26^ and non-statin lipid lowering medication. Differences in associations among subgroups were evaluated by including interaction terms between statin and each variable. The definitions of covariates are provided in Table S4.

Several sensitivity analyses were performed to evaluate the robustness of the findings, with their detail available in the Supplementary Result. A sensitivity analysis was performed using a change in Low Density Lipoprotein Cholesterol (LDL-C) as positive control and withdrawal from the study as a negative control to validate the study design in identifying an expected effect. A positive control was employed to validate whether the study's design and analytical methods could correctly identify the expected effect, while a negative control was used to identify confounding variables that might falsely indicate an effect on our primary outcome. To account for potential differences in the comparator group, we excluded participants with LDL-C levels exceeding 160 mg/dL, and those with Total Cholesterol (TC) levels of 290 mg/dL or higher, fitting different models for each. We repeated the main analysis excluding other lipid-modifying agents (e.g., ezetimibe and omega-3 fatty acids). To address potential immortal time bias from hierarchical statin initiation, in addition to TD analysis, we performed a time-matched nested case–control approach where we matched each case to one control (1:1 ratio) based on time since ASPREE randomisation, following IPTW to adjust for all included confounders in the main analysis to maintain a covariate balance.^24^ Finally, E-values were calculated to assess the robustness of our findings to unmeasured confounding.

### Ethics approval and informed consent

This study was a secondary analysis of data from the ASPREE trial and its longitudinal extension, ASPREE-XT, and was conducted in accordance with the Declaration of Helsinki 1964, as revised in 2008. The ASPREE ethics approval was obtained from Monash University Human Research Ethics Committee (IRB00002519; 2006/745 MC), the Royal Australasian College of General Practitioners Ethics Committee (NREEC 02/22b), Human Research Ethics Committee (Tasmanian) Network (H0008933), Goulburn Valley Health Ethics & Research Committee (GVH-21/07), and ACT Health Human Research Ethics Committee (11/07.997). In the United States, all ASPREE sites obtained Institutional Review Board (IRB) approval from their respective institutions.^16^ Oversight of ASPREE-XT was maintained by the same ethics committees. In the USA, the ASPREE-XT study was overseen by a single central IRB at the University of Iowa (IRB ID #201904807). All participants provided written informed consent prior to enrollment, including consent for future research use of their data.

### Role of the funding source

The funder of the study had no role in the study design, data collection, data analysis, data interpretation, or writing of the report. GRD, MM, and ML had full access to and verified the study data, and GRD had final responsibility for the decision to submit the manuscript for publication.

## Results

**Fig. 1:**
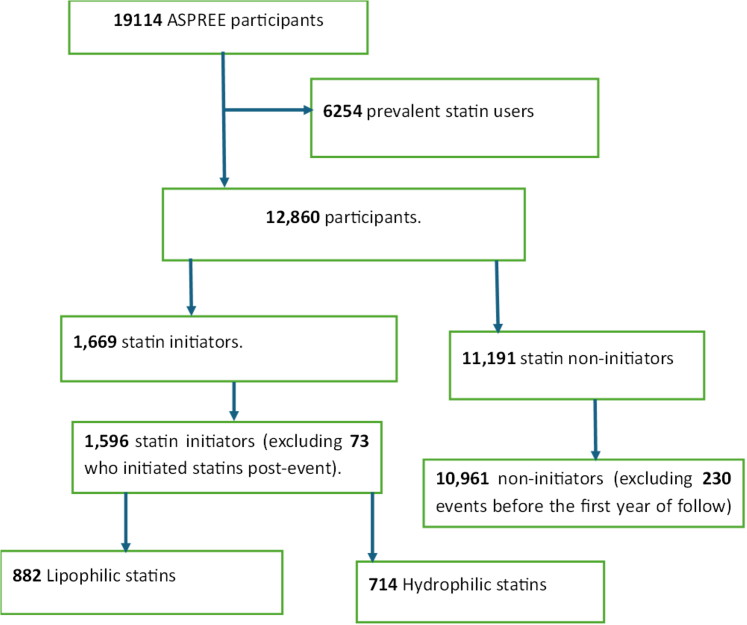
CONSORT diagram for study participants.

**Fig. 2:**
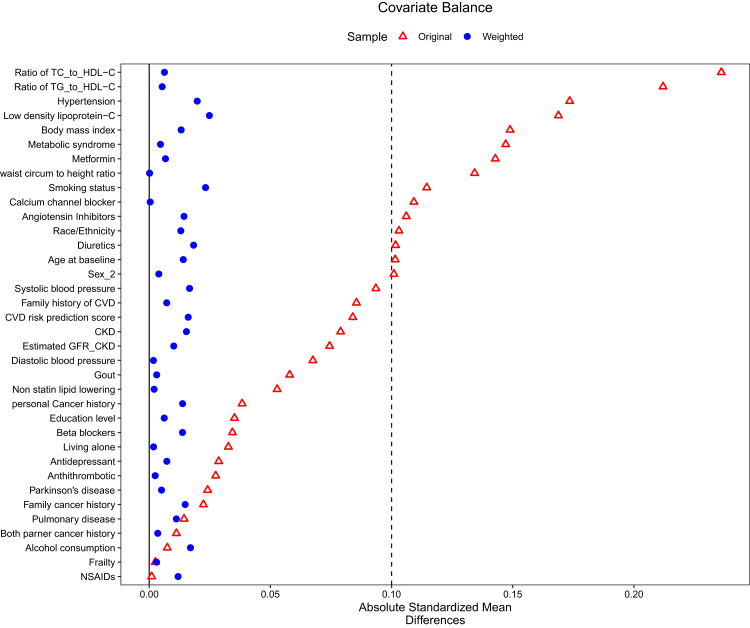
Standardized mean difference for pair-wise comparisons of characteristics before and after weighting (statin versus no statin). A weighted value below the dotted line or 0.1 indicates good balance between the groups. Abbreviation: CKD, Chronic Kidney Disease; CVD, Cardiovascular Disease; GFR, Glomerular Filtration Rate; HDL-C, High Density Lipoprotein Cholesterol; NSAIDs, Non-Steroidal Anti-Inflammatory Drugs; TC, Total Cholesterol; TG, Triglyceride. Both partner cancer history refers to cases where both the participant's mother and father have a documented history of cancer.

A total of 19,114 participants were recruited from March 01, 2010, to December 31, 2014, with follow-up through June 12, 2017, for the ASPREE trial period and continued follow-up until January 08, 2022, for the observational study (ASPREE-XT). The final analysis included 12,557 participants, including 1596 statin and 10,961 non-initiators (Fig. 1). The mean (SD) age was 75.2 (4.6) years, and 6887 participants (54.8%) were female. Atorvastatin was the most prescribed statin, followed by rosuvastatin and simvastatin (Table S6). The characteristics of statin and non-statin initiator groups before and after applying IPTW are shown in Table 1. Significant differences were observed between the two groups prior to weighting (Figure S3). Before weighting, the statin initiators were younger, more often in the obese than normal BMI range, had a higher percentage of current smokers and a family history of CVD. Statin initiators were also more likely to have comorbidities such as HTN, DM, Parkinson's disease, metabolic syndrome, chronic kidney disease (CKD) and a high CVD risk prediction score, and elevated ratios of TC to HDL-C and LDL-C levels compared to non-initiators. Statin initiators were also more likely to receive renin angiotensin inhibitors, diuretics, calcium channel blockers, and metformin. After applying IPTW, all characteristics were well balanced between groups, with all standardised mean differences in pairwise comparisons less than 0.1 (Fig. 2) and the distribution of weights are summarised in Table S7.

**Table 1:** Demographic and health characteristics before and after inverse probability weighting for the study population stratified by statin use.

	Before IP weighting			After IP weighting		
	Statin initiators (N = 1596)	Non-statin initiators (N = 10,961)	Total (N = 12,557)	Statin initiators (N = 1587)	Non-statin initiators (N = 10,961)	Total (N = 12,549)
Age: means (SD) and n (%)						
Mean (SD)	74.8 (4.5)	75.2 (4.6)	75.2 (4.6)	75.2 (4.7)	75.2 (4.6)	75.2 (4.6)
65–69	66 (4.1)	281 (2.6)	347 (2.8)	44 (2.8)	304 (2.8)	349 (2.8)
70–74	898 (56.3)	6088 (55.5)	6986 (55.6)	868 (54.7)	6094 (55.6)	6962 (55.5)
75–79	415 (26.0)	2831 (25.8)	3246 (25.9)	421 (26.5)	2836 (25.9)	3257 (26.0)
80–84	173 (10.8)	1289 (11.8)	1462 (11.6)	190 (12.0)	1277 (11.6)	1467 (11.7)
≥85	44 (2.8)	472 (4.3)	516 (4.1)	64 (4.0)	450 (4.1)	514 (4.1)
Gender (female)	800 (50.1)	6087 (55.5)	6887 (54.8)	862 (54.3)	6018 (54.9)	6880 (54.8)
Education- n (%)						
9–11	721 (45.2)	4763 (43.5)	5484 (43.7)	696 (43.8)	4787 (43.7)	5483 (43.7)
12–15	486 (30.5)	3165 (28.9)	3651 (29.1)	462 (29.1)	3184 (29.1)	3646 (29.1)
15+	389 (24.4)	3033 (27.7)	3422 (27.3)	430 (27.1)	2990 (27.3)	3420 (27.3)
Race/Ethnicity n (%)						
Black American	107 (6.7)	443 (4.0)	550 (4.4)	73 (4.6)	483 (4.4)	556 (4.4)
Latino	44 (2.8)	231 (2.1)	275 (2.2)	36 (2.3)	240 (2.2)	276 (2.2)
White	1421 (89.0)	10,161 (92.7)	11,582 (92.2)	1460 (92.0)	10,108 (92.2)	11,568 (92.2)
Others	24 (1.5)	126 (1.1)	150 (1.2)	18 (1.1)	130 (1.2)	148 (1.2)
Smoking status n (%)						
Current smoker	79 (4.9)	386 (3.5)	465 (3.7)	55 (3.4)	404 (3.7)	458 (3.7)
Former smoker	699 (43.8)	4363 (39.8)	5062 (40.3)	630 (39.7)	4415 (40.3)	5045 (40.2)
Never smoker	818 (51.3)	6212 (56.7)	7030 (56.0)	903 (56.9)	6142 (56.0)	7045 (56.1)
Living Alone-n (%)	494 (31.0)	3565 (32.5)	4059 (32.3)	512 (32.3)	3545 (32.3)	4057 (32.3)
Alcohol consumption n (%)						
Current	1230 (77.1)	8500 (77.5)	9730 (77.5)	1220 (76.9)	8491 (77.5)	9712 (77.4)
Former	115 (7.2)	600 (5.5)	715 (5.7)	87 (5.5)	623 (5.7)	710 (5.7)
Never	251 (15.7)	1861 (17.0)	2112 (16.8)	280 (17.6)	1847 (16.9)	2127 (16.9)
Family history of CVD n (%)	1003 (62.8)	6433 (58.7)	7436 (59.2)	947 (59.6)	6491 (59.2)	7438 (59.3)
Family cancer history n (%)	948 (59.4)	6634 (60.5)	7582 (60.4)	968 (61.0)	6620 (60.4)	7588 (60.5)
Personal cancer history n (%)	287 (18.0)	2113 (19.3)	2400 (19.1)	314 (19.8)	2095 (19.1)	2409 (19.2)
Body mass index in kg/m^2^- n (%)						
<25	392 (24.6)	3312 (30.2)	3704 (29.5)	479 (30.2)	3238 (29.5)	3716 (29.6)
25–30	704 (44.1)	4829 (44.1)	5533 (44.1)	690 (43.4)	4828 (44.0)	5518 (44.0)
≥30	500 (31.3)	2820 (25.7)	3320 (26.4)	419 (26.4)	2896 (26.4)	3315 (26.4)
LDL-C categories in mg/dL						
≥100	1421 (89.0)	9126 (83.3)	10,547 (84.0)	1348 (84.9)	9206 (84.0)	10,554 (84.1)
<100	175 (11.0)	1835 (16.7)	2010 (16.0)	239 (15.1)	1756 (16.0)	1995 (15.9)
Comorbidities n (%)						
Hypertension	1234 (77.3)	7626 (69.6)	8860 (70.6)	1136 (71.6)	7733 (70.5)	8869 (70.7)
Diabetes mellitus	182 (11.4)	595 (5.4)	777 (6.2)	144 (9.1)	635 (5.8)	779 (6.2)
Metabolic syndrome	438 (27.4)	2314 (21.1)	2752 (21.9)	350 (22.0)	2400 (21.9)	2750 (21.9)
Chronic kidney disease	301 (18.9)	1725 (15.7)	2026 (16.1)	268 (16.9)	1767 (16.1)	2036 (16.2)
Gout	75 (4.7)	385 (3.5)	460 (3.7)	57 (3.6)	401 (3.7)	458 (3.6)
Pulmonary disease	215 (13.5)	1431 (13.1)	1646 (13.1)	210 (13.2)	1438 (13.1)	1648 (13.1)
Frailty n (%)						
Pre frail	616 (38.6)	4195 (38.3)	4811 (38.3)	603 (38.0)	4211 (38.4)	4815 (38.4)
Frail	33 (2.1)	235 (2.1)	268 (2.1)	30 (1.9)	236 (2.2)	266 (2.1)
Parkinson's disease	24 (1.5)	135 (1.2)	159 (1.3)	19 (1.2)	139 (1.3)	158 (1.3)
Gout	433 (27.1)	2757 (25.2)	3190 (25.4)	420 (26.4)	2773 (25.3)	3193 (25.4)
CVD risk prediction n (%)						
Low risk	992 (62.2)	7366 (67.2)	8358 (66.6)	1043 (65.7)	7299 (66.6)	8343 (66.5)
Moderate risk	489 (30.6)	2857 (26.1)	3346 (26.6)	428 (26.9)	2917 (26.6)	3345 (26.7)
High risk	115 (7.2)	738 (6.7)	853 (6.8)	116 (7.3)	745 (6.8)	861 (6.9)
Continuous clinical factors –mean (SD)						
Ratio of TC to HDL-C level	4.0 (1.1)	3.6 (1.2)	3.7 (1.2)	3.8 (1.0)	3.7 (1.2)	3.7 (1.2)
Ratio of TG to HDL-C level	2.5 (2.0)	2.1 (1.7)	2.1 (1.8)	2.2 (1.8)	2.1 (1.8)	2.1 (1.8)
Ratio of waist circumference to height	0.6 (0.1)	0.6 (0.1)	0.6 (0.1)	0.6 (0.1)	0.6 (0.1)	0.6 (0.1)
Systolic blood pressure mmHg	141.0 (16.7)	139.0 (16.6)	139.2 (16.7)	140.0 (16.9)	139.2 (16.6)	139.3 (16.7)
Diastolic blood pressure mmHg	78.3 (10.0)	77.4 (10.0)	77.5 (10.0)	77.7 (9.9)	77.5 (10.0)	77.5 (10.0)
Estimated Glomerular filtration rate	72.5 (14.3)	73.6 (13.5)	73.4 (13.6)	73.3 (13.9)	73.4 (13.6)	73.4 (13.7)
Medications- n (%)						
Non-statin lipid lowering medication	88 (5.5)	481 (4.4)	569 (4.5)	85 (5.3)	485 (4.4)	570 (4.5)
Antithrombotic	108 (6.8)	665 (6.1)	773 (6.2)	97 (6.1)	673 (6.1)	770 (6.1)
Metformin	73 (4.6)	222 (2.0)	295 (2.3)	39 (2.4)	258 (2.4)	297 (2.4)
NSAIDs	279 (17.5)	1916 (17.5)	2195 (17.5)	269 (17.0)	1915 (17.5)	2184 (17.4)
Diuretics	355 (22.2)	1987 (18.1)	2342 (18.7)	310 (19.5)	2046 (18.7)	2356 (18.8)
Angiotensin inhibitors (ACEIs/ARBs)	428 (26.8)	2429 (22.2)	2857 (22.8)	371 (23.3)	2493 (22.7)	2864 (22.8)
Calcium channel blockers	298 (18.7)	1600 (14.6)	1898 (15.1)	241 (15.2)	1657 (15.1)	1898 (15.1)
Beta blockers	125 (7.8)	760 (6.9)	885 (7.0)	120 (7.6)	774 (7.1)	895 (7.1)
Antidepressant	186 (11.7)	1185 (10.8)	1371 (10.9)	177 (11.1)	1199 (10.9)	1376 (11.0)

ACEIs, Angiotensin-Converting Enzyme Inhibitors; ARBs, Angiotensin II Receptor Blockers; BMI, Body mass index; CVD, Cardiovascular disease; eGFR, estimated HDL-C: High-density lipoprotein cholesterol; LDL-C, Low-density lipoprotein cholesterol; NSAIDs, Non-steroidal anti-inflammatory drugs; TG, Triglyceride; TC, Total cholesterol.

**Fig. 3:**
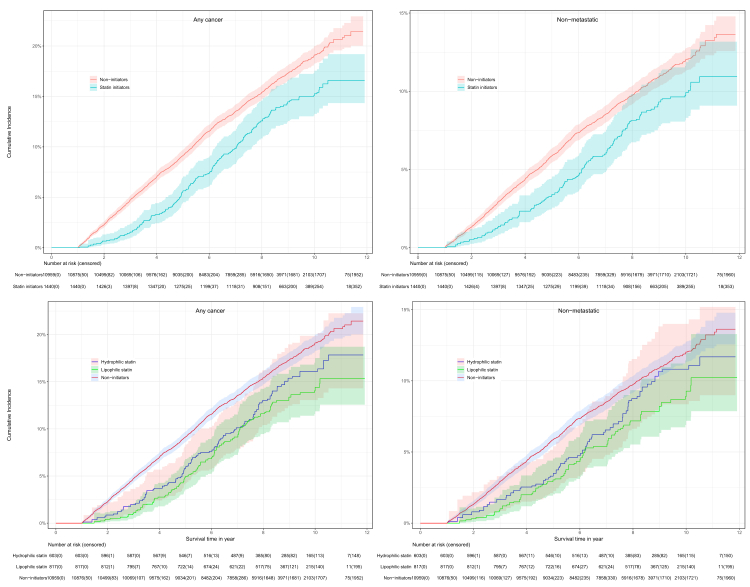
Cumulative incidence curves for statin initiators versus non-statin initiators for overall (any cancer) and non-metastatic cancer events.

**Fig. 4:**
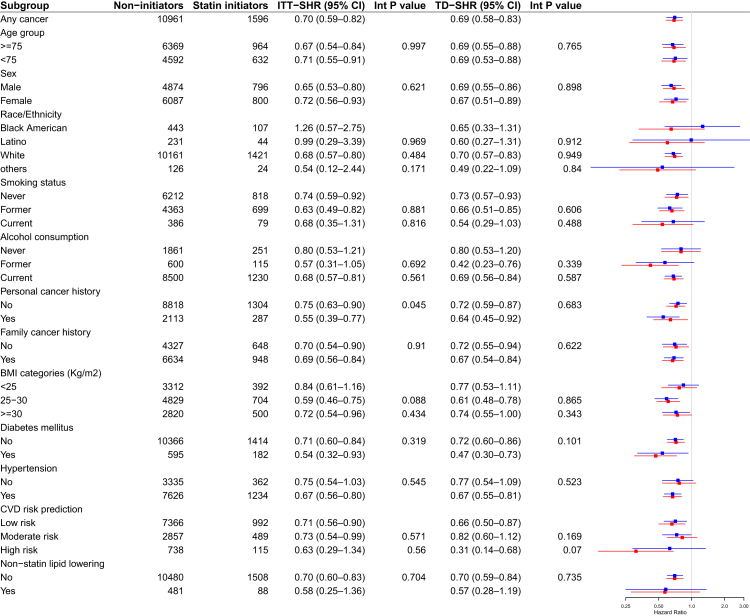
Subgroup analysis for statin versus non-statin initiators groups based on important baseline characteristics (red represents TD, and blue represents ITT). Abbreviation: BMI, Body Mass Index; CVD, Cardiovascular Disease; Int P value, Interaction P value; TD-SHR, Time-Dependent Sub-distribution Hazard Ratio; ITT-SHR, Intention-to-Treat Sub-distribution Hazard Ratio (time-fixed analysis).

After a median follow-up of 8.3 years (Interquartile range (IQR); 6.5–9.5), 210 statin initiators developed cancer (16.0 per 1000 person years (PY), 95% CI: 13.8–18.3) while 1793 statin non-initiators developed cancer (21.6 per 1000 PY, 95% CI: 20.6–22.6). In both ITT and TD analyses, statin initiation was associated with a lower risk of any cancer (ITT-SHR: 0.70 [0.59–0.82]; TD-SHR: 0.69 [0.58–0.83]), non-metastatic (0.71 [0.58–0.87]; 0.69 [0.55–0.86]) and metastatic (0.70 [0.52–0.93]; 0.74 [0.55–1.00]) cancers (Table 2, Fig. 3). There was no significant heterogeneity in the associated effect of statins across all baseline subgroups considered (Fig. 4, all p-value for interaction >0.05). Over a median follow-up of 8.3 years, the estimated NNT with statins to prevent one cancer event was 31 (95% CI: 25–47) for any cancer, 56 (95% CI: 44–87) for metastatic cancer, and 72 (95% CI: 46–100) for non-metastatic cancer. These findings indicate that statin therapy may confer a greater absolute benefit in reducing overall cancer incidence compared to specific subtypes, with higher NNTs observed for metastatic and non-metastatic cancers.

**Table 2:** Cumulative incidence of cancer and association with statin use under time fixed (intention-to-treat) and time-dependent analyses.

	Number of events (incidence rate-IR per 1000 person years [95% CI])		SHR (robust 95% CI)		P value^a^
	Statin initiators (1596)	Non statin initiators (10,961)	Time fixed (ITT)	Time updated/dependent	
Any cancer	210 (16.0 [13.8–18.3])	1793 (21.6 [20.6–22.6])	0.70 (0.59–0.82)	0.69 (0.58–0.83)	0.0001
Non metastatic	141 (10.3 [8.5–12.1])	1121 (13.5 [12.7–14.3])	0.71 (0.58–0.87)	0.69 (0.55–0.86)	0.004
Metastatic	69 (5.7 [4.3–7.0])	672 (8.0 [7.4–8.6])	0.70 (0.52–0.93)	0.74 (0.55–1.00)	0.027
Incident metastatic	38 (3.0 [2.0–4.0])	347 (4.1 [3.7–4.6])	0.74 (0.50–1.07)	0.78 (0.52–1.17)	0.21
Recurrent	9 (0.9 [0.4–1.4])	89 (1.1 [0.8–1.3])	0.59 (0.28–1.21)	0.71 (0.34–1.41)	0.17
Site-specific cancers					
Bladder	7 (0.51 [0.10–0.92])	68 (0.83 [0.63–1.02])	0.64 (0.29–1.44)	0.97 (0.40–2.32)	0.62
Brain	3 (0.25 (0.00–0.53])	20 (0.23 [0.13–0.34])	–	–	–
Breast	20 (1.68 [0.94–2.42])	206 (2.44 [2.11–2.78])	0.55 (0.33–0.93)	0.32 (0.16–0.64)	0.01
Cervical	0	2	–	–	–
Colon	34 (2.56 [1.65–3.47])	219 (2.62 [2.27–2.97])	0.85 (0.56–1.29)	0.88 (0.55–1.41)	0.88
Gallbladder	1 (0.05 [0.00–0.17])	19 (0.22 [0.12–0.33])	–	–	
Kidney	4 (0.21 [0.00–0.48])	35 (0.43 [0.29–0.57])	0.59 (0.15–2.43)	1.05 (0.23–4.90)	0.89
Liver	0	9 (0.11 [0.04–0.18])	–	–	
Lung	20 (1.34 [0.68–2.00])	132 (1.61 [1.33–1.88])	0.86 (0.52–1.42)	1.15 (0.68–1.94)	0.88
Melanoma	19 (1.34 [0.68–2.00])	177 (2.11 [1.80–2.42])	0.66 (0.39–1.11)	0.62 (0.33–1.16)	0.15
Ovarian	8 (0.73 [0.25–1.22])	56 (0.67 [0.49–0.84])	1.27 (0.56–2.89)	1.25 (0.51–3.06)	0.88
Pancreas	8 (0.66 [0.20–0.13])	50 (0.60 [0.43–0.76])	0.91 (0.40–2.09)	1.04 (0.45–2.38)	0.89
Prostate	44 (3.32 [2.28–4.36])	332 (4.04 [3.61–4.47])	0.67 (0.47–0.95)	0.56 (0.38–0.81)	0.027
Gastric	5 (0.36 [0.02–0.71])	72 (0.87 [0.67–1.07])	0.42 (0.16–1.08)	0.73 (0.26–2.06)	0.35
Thyroid	3 (0.15 [0.00–0.38])	9 (0.12 [0.04–0.19])	–	–	–
Unknown primary	1 (0.08 [0.00–0.25])	38 (0.45 [0.31–0.60])	–	–	–
Others	11 (1.00 [0.43–1.57])	112 (1.34 [1.09–1.59])	0.82 (0.43–1.55)	0.77 (0.40–1.48)	0.25
**Association between statin use and risk of cancer stratified by statin lipophilicity**					
		Treatment strategy	Number of events (IR per 1000 PY [95% CI])	SHR (robust 95 CI)	
Any cancer		Lipophilic	109 (15.3 [12.2–18.5])	0.64 (0.51–0.79)	0.008
		Hydrophilic	101 (17.8 [13.9–21.6])	0.78 (0.63–0.97)	0.06
Non metastatic cancer		Lipophilic	72 (9.5 [7.0–12.0])	0.64 (0.49–0.84)	<0.0001
		Hydrophilic	69 (11.7 [8.6–14.8])	0.80 (0.61–1.06)	0.062
Metastatic cancer		Lipophilic	37 (5.8 [3.9–7.7])	0.65 (0.45–0.94)	0.006
		Hydrophilic	32 (6.1 [3.8–8.3])	0.77 (0.52–1.13)	0.064

Other cancers: Anal, head and neck, mesothelioma, neuroendocrine, other genitourinary, skin (excluding non-melanoma skin cancer), sarcoma, esophageal or esophageal–gastric junction cancer, gastrointestinal, and various other cancers. ITT, Intention to treat analysis; CI, Confidence Interval; SHR, Sub-distribution Hazard Ratio.

The analysis of statin types (lipophilic or hydrophilic) was conducted only using a time-fixed (ITT) approach.

a P-values were adjusted for multiple comparisons using the Benjamini–Hochberg procedure, based on intention-to-treat analyses for site-specific cancers and for analyses stratified by statin lipophilicity.

In site-specific analyses of common cancer types, statin use was associated with statistically significant lower risk of breast cancer (ITT-SHR: 0.55 [95% CI 0.33–0.93]; TD-SHR: 0.32 [95% CI 0.16–0.64]) and prostate cancer (ITT-SHR: 0.67 [95% CI 0.47–0.95]; TD-SHR: 0.56 [95% CI 0.38–0.81]) compared to those who did not initiate statin use. For other common cancers, such as colon, lung, and melanoma, the observed differences were not statistically significant (Table 2).

A secondary analysis compared lipophilic statin initiators and hydrophilic statin initiators with non-initiators. The baseline characteristics of the study participants before and after weighing were summarised for statin types (lipophilic and hydrophilic) and non-initiators in Table S8 and all SMD were <0.1 (Figure S4). Compared with non-initiation, use of a lipophilic statin was associated with a lower risk of non-metastatic (0.64 [0.49–0.84]) and metastatic (0.65 [0.45–0.94]) cancers, while hydrophilic statin initiation was not (Table 2).

The results were robust to several sensitivity analyses (Table S9). Statin initiation was associated with a change in mean serum LDL-C levels, the designated positive control by 30.3 mg/dL (approximately 1 mmol/L; P < 0.001). The negative control analysis showed no significant difference in withdrawal rates between statin initiators and non-initiators. Together, these two sensitivity analyses strengthen confidence in the main findings, suggesting they are unlikely to be influenced by unmeasured confounding or prevalent user bias. After excluding participants with high serum LDL-C and TC, or other lipid-lowering medication, the results remain unchanged. Analysis using a time-matched nested case–control also yielded results similar to the main analyses, suggesting that the main analysis finding is less likely to be affected by immortal time bias. Finally, the E value for any cancer was 1.88, indicating unmeasured confounders would need to have a collective risk ratio of at least 1.88 with exposure and cancer to fully account for the estimated association (Figure S5).

## Discussion

Using data from a large-scale, multicenter RCT of community-dwelling older adults in Australia and the United States, we emulated a target trial to assess the association between statin initiation and risk of cancer events. We examined whether these effects differed by statin lipophilicity. Statin use was significantly associated with a lower risk of any cancer, with consistent protective associations observed for non-metastatic (NNT = 72) and metastatic (NNT = 56) cancers. The magnitude of these associations varied according to statin lipophilicity, suggesting potential pharmacokinetic influences on cancer incidence.

Several observational studies^9^, ^10^, ^11^, ^12^ and an RCT study^27^ have reported a potential protective association between statin use and cancer risk. These findings contrast with two major meta-analyses of RCTs, which showed no link between statin use and cancer risk.^28^^,^^29^ This discrepancy could be due to differences in study population, duration of follow up and outcome definition. The trials synthesised in those meta-analyses were primarily cardiovascular studies enrolling middle-aged participants and relatively short follow-up periods of three to five years. These designs are well suited for detecting cardiovascular benefits but less capable of cancer outcomes, which often involve long latency periods. In contrast, ASPREE enrolled healthy older adults with a mean age of 75 years and a follow-up of up to 12 years. This demographic has a substantially higher risk of cancer, and biological processes such as chronic inflammation may amplify the pleiotropic effects of statins.

Differences in outcome ascertainment may also explain this inconsistent evidence. In ASPREE, cancer diagnoses were adjudicated by two blinded experts, with discrepancies resolved by a third reviewer. This level of precision contrasts with most cardiovascular trials, where cancer is typically recorded as an adverse event without formal adjudication, increasing the risk of misclassification. Nevertheless, our study is observational, and residual confounding due to unmeasured lifestyle factors may contribute to the observed association, despite extensive adjustment and sensitivity analyses. ASPREE participants were generally healthy older adults, and those with pre-existing conditions may exhibit different responses to statin therapy.^30^ These limitations underscore the need for caution in interpreting causality. Yet, the consistency of our findings across sensitivity analyses and all subgroups suggests that the benefits of statin therapy may extend beyond cardiovascular prevention, potentially influencing pathways relevant to cancer development. Definitive clarification will require randomised trials specifically designed to evaluate cancer outcomes in older adults with adequate long-term follow-up and rigorous endpoint adjudication.

Statins may exert anticancer effects through two mechanisms, including cholesterol-mediated and non-cholesterol-mediated, which are also called pleiotropic effects. The cholesterol-mediated includes the inhibition of the mevalonate pathway, where statins block HMG-CoA reductase, disrupting the synthesis of isoprenoids necessary for the activation of small GTPases (e.g., Ras and Rho), reducing cancer cell proliferation; and disruption of lipid rafts, where lowering cholesterol levels impairs the function of proteins involved in cell signaling, adhesion, and migration, thus limiting cancer cell invasion. The non-cholesterol-mediated mechanisms include: (i) induction of apoptosis and cell cycle arrest, where statins promote cancer cell death by activating apoptotic pathways and downregulating cyclins and CDKs; (ii) anti-inflammatory and immunomodulatory effects, where statins reduce pro-inflammatory cytokines (e.g., TNF-α, IL-6), C-reactive protein (CRP) and enhance immune responses, diminishing tumor-supportive inflammation.^3^ Other mechanisms may involve disruption of lipid rafts on cell membranes, downregulation of matrix metalloproteinases, and limiting the acquisition of migratory and invasive properties by cancer cells.^31^ These effects support the role of statins not only in reducing primary cancer risk but also in inhibiting metastasis, underscoring their potential for comprehensive cancer prevention.

Our findings indicate that statin initiation in older adults is associated not only with a lower overall cancer incidence but also with a reduced risk of metastatic disease, raising the possibility that statins may influence cancer progression. This observation is consistent with evidence from a comprehensive meta-analysis of randomised controlled trials and observational studies involving 163,005 participants, which reported improved progression-free survival in advanced-stage cancer among statin users (HR 0.76; 95% CI 0.65–0.87).^32^ Statins inhibit cholesterol synthesis by targeting HMG-CoA reductase, the key intermediates for cell proliferation, membrane integrity, and signalling processes essential for tumour growth and metastasis.^33^ Disruption of these downstream products interferes with cancer cell survival, delays progression, and suppresses metastatic potential. Statins have also been associated with the cessation of cell-cycle progression in cancer cells, resulting in antiproliferative effects, suppression of key cellular functions, and increased sensitivity to radiation therapy.^34^ Indeed, rapidly growing tumours require a high absorption of extracellular cholesterol, and statin-induced reductions in circulating or locally produced cholesterol levels may prevent metastasis cancer.

We observed site-specific differences in cancer risk following statin initiation, with a significant association for breast and prostate cancers. These patterns are consistent with evidence that statins may exert stronger effects in hormone-sensitive malignancies.^5^^,^^35^ In breast cancer, statins may inhibit tumour progression primarily by suppressing proliferation and inducing apoptosis in mammary carcinoma cells through inhibition of the mevalonate pathway.^5^ For prostate cancer, statins may disrupt lipid rafts, which impairs key signalling pathways including the androgen receptor (AR), EGFR, and PI3K–AKT, thereby reducing tumour growth.^36^ Additionally, statins lower cholesterol and androgen synthesis, inhibit Ras/Rho GTPase prenylation, and block dehydroepiandrosterone sulphate uptake via the SLCO2B1 transporter, further limiting tumour progression.^6^

Our analysis revealed that both lipophilic and hydrophilic statins were associated with a lower risk of overall cancer events, although the association was more pronounced for lipophilic statins. However, while lipophilic statin use remained significantly associated with both metastatic and non-metastatic cancers, hydrophilic statins showed no such association. This variation reflects differences in pharmacokinetic properties. Lipophilic statins can passively diffuse across cell membranes, achieving higher intracellular concentrations in extrahepatic tissues and tumour cells compared to hydrophilic statins, which rely on active hepatic transport and have limited tumour penetration.^37^ This enhanced bioavailability allows lipophilic statin to more effectively inhibit the mevalonate pathway, reducing prenylation of oncogenic proteins (e.g., Ras, Rho), disrupting proliferative and survival signalling, and inducing apoptosis. In metastatic cancer, lipophilic statins may also inhibit epithelial–mesenchymal transition, maintain tumour cell dormancy, and impair metastatic outgrowth—mechanisms that are less accessible to hydrophilic statins due to their restricted tissue distribution.^38^

This study's strengths lie in its large sample size, long duration of follow-up, and methodological rigour. Statin use was determined by clinical indications, reflecting real-world decision-making and enhancing the applicability of the findings to clinical practice. Using a target trial emulation framework helped mitigate potential confounding by providing an unbiased estimation. The study's robustness is further supported by the formal and rigorous adjudication of cancer outcomes, ensuring accuracy and reliability, and the targeting of an older age cohort at greatest risk of cancer. Unlike most comparisons of statin initiators versus non-initiators, this study examined differences between lipophilic and hydrophilic statins, uncovering potential variations in their anticancer effects. Certain limitations should be acknowledged. First, in the absence of randomisation to treatment, the possibility of residual confounding due to unmeasured factors and immortal time bias cannot be entirely ruled out despite rigorous efforts to mitigate them. Second, while this study assessed the potential effect of lipophilicity, the influence of dose, potency and adherence could not be explored. Participants were relatively healthy older adults, which limits the generalizability of the findings to populations with multiple comorbidities. The estimates of the NNT are derived from a prospective cohort study rather than an RCT, and therefore, they should be interpreted with caution. Finally, the relatively small number of events in subgroup and site-specific analyses might impact statistical power. Future research should consider these aspects through long-term, large-scale RCTs of the older adult population.

In this target trial emulation, statin initiation was significantly associated with a lower risk of cancer, including metastatic and non-metastatic events in older people. However, while this association had remained for lipophilic statins in both metastatic and non-metastatic cancer, no comparable pattern was observed for hydrophilic statins. Despite the use of advanced statistical adjustments and a target trial emulation design, the inherent potential for residual confounding in the observational study design warrants cautious interpretation of the findings.

## Contributors

GRD, ML and MM had full access to and verified the data of the study. MM is a senior author. **GRD:** Conceptualisation, Methodology, Software, Data curation, Formal analysis and writing the original draft. **ML& ND:** Software, Methodology, Writing, review, and editing. **MB & RW:** Conceptualisation, Writing-review and editing,: RW, JJM, AMT, and RLW: data curation and investigation: GRD, AMT, PG, ZZ, RLW, SGO, ASE, AMM, MN, JLM, ARK, WLO, CMR, RCS, AC, DCC, SZ and JJM: Writing-review and editing, **MM:** Conceptualisation, Methodology, Supervision, writing the original draft, Writing-review and editing.

## Data sharing statement

The de-identified dataset used in this study can be made available to researchers upon submission of an Expression of Interest (EOI) outlining the research objectives and methodology, subject to approval by the ASPREE Executive Committee. Detailed access procedures can be found at: http://ams.aspree.org. Software codes for reproducing the results can be made available to researchers upon request.

## Declaration of interests

**MB** reports support for the current manuscript through an NHMRC Leadership 3 Investigator Grant (GNT2017131). He has also received grants from the National Health and Medical Research Council (Australia), Principal Research Fellowship, Psychscene.com, WFSBP, NeuroSAS, CINP, Shanghai Mental Health Center, Penn State College of Medicine, Baszucki Brain Research Fund, Danmarks Frie Forskningsfond, Psykiatrisk Center København, and Controversias Psiquiatria Barcelona, with payments made directly to Professor Berk. Additional funding was received from MRFF, Wellcome Trust, Victorian Government Department of Jobs, Precincts and Regions, CDMRP (USA), Janssen Lundbeckfonden Copenhagen, St. Biopharma, AEDRTC, PCORI, Stanley Medical Research Institute, Victorian Medical Research Acceleration Fund, CRE, and the Victorian COVID-19 Research Fund, with payments directed to his institution.

MB also reports receiving royalties or license fees from Cambridge University Press and Allen & Unwin, and consulting fees from Allergan, AstraZeneca, Bioadvantex, Bionomics, Collaborative Medicinal Development, Janssen, Lundbeck, Merck, Otsuka, Servier, and the Milken Foundation. He has received honoraria or payments from the Global Congress of Biological Psychiatry (India), Otsuka CNS, Eisai Australia, RANZCP (New Zealand), Sandoz, Allori, Lundbeck, the World Congress of Psychiatry, the African College of Neuropsychopharmacology, and the SVI Inaugural Health Matters Webinar Series. He also discloses support for travel and/or conference attendance from Medisquire India and the 25th International Symposium on Current Issues in Psychiatry. Professor Berk holds issued patents related to the modulation of physiological processes and agents useful for same, as well as for the modulation of central nervous system diseases and related disorders. He serves as an Advisory Board Member for *Thieme Pharmacopsychiatry*, a Research Advisory Committee Member for Beyond Blue, and an Advisory Council Member for the Australian Early Psychosis Collaborative Consortium (AEPCC).

**SZ** reports receiving grant funding from the NHMRC (Australian Government Department of Health and Aged Care), JDRF Centre of Excellence, Eli Lilly Australia, Boehringer-Ingelheim, CSL Seqirus, AstraZeneca, Novo Nordisk, and Moderna. These funds were paid to Monash University. She also discloses her role as a Board Director for the Australian Clinical Trials Alliance.

**RCS** reports serving as a site principal investigator for the PREVENTABLE randomised clinical trial (NCT04262206), funded by the U.S. National Institute on Aging (U19AG065188). The trial involves statin randomisation to assess disability-free longevity. This work is unrelated to the content of the current manuscript.

GRD, ND, ML, AMT, PG, ZZ, RW, RLW, SGO, ASE, AMM, MN, JLM, ARK, WLO, CMR, AC, DCC, JJM, and MM declare no competing interests.
