# Supplementary material for: Association between statin use and risk of incident cancer in healthy older adults: a target trial emulation using data from a multicentre, randomised trial of community-dwelling older adults in Australia and the USA
Source: eClinicalMedicine. 2026 Jan 12;91:103746. doi: 10.1016/j.eclinm.2025.103746 (PMC12828538; doi:10.1016/j.eclinm.2025.103746)
Supplement: Supplementary Material [file mmc1.docx]

**Association between statin use and risk of incident cancer in healthy older adults: a target trial emulation using data from a multicentre, randomised trial of community-dwelling older adults in Australia and the USA**

**Contents**

[List of Supplementary Tables 1](#_Toc215052648)

[List of Supplementary Figures 2](#_Toc215052649)

[Graphical Abstract 3](#_Toc215052650)

[Supplementary Methods 3](#_Toc215052651)

[Protocol of the hypothetical target trial emulation 3](#_Toc215052652)

[Cancer Definition 6](#_Toc215052653)

[Study’s Power and detectable effect size 7](#_Toc215052654)

[Inverse Probability Weighting 9](#_Toc215052655)

[Confounder selection 10](#_Toc215052656)

[Directed Acyclic Graph 10](#_Toc215052657)

[Proportional Hazard Assumption 11](#_Toc215052658)

[Supplementary Results 13](#_Toc215052659)

[Distribution of individual statin medications 13](#_Toc215052660)

[Weight distribution and covariate balance plot 13](#_Toc215052661)

[Secondary analysis (lipophilic vs hydrophilic; vs no statin) 15](#_Toc215052662)

[Sensitivity analysis 19](#_Toc215052663)

[Missing data 22](#_Toc215052664)

[Reference 23](#_Toc215052665)

## List of Supplementary Tables

[Table S1: Specification (protocol) of the target trial and its emulation based on TARGET guideline 3](#_Toc217646893)

[Table S2. Cancer diagnosis criteria used in ASPREE 6](#_Toc217646894)

[Table S3: Missing baseline covariate data among statin initiators and non-initiators 7](#_Toc217646895)

[Table S4: Detectable effect size at 80% of the study and observed effect size. 8](#_Toc217646896)

[Table S5: Definition of covariates. 8](#_Toc217646897)

[Table S6: Distribution of individual statin medications 13](#_Toc217646898)

[Table S7: Distribution of inverse probability treatment weights 14](#_Toc217646899)

[Table S8: Demographic and health characteristics before and after weighting for statin types (lipophilic vs hydrophilic) vs. no statin. 15](#_Toc217646900)

[Table S9: Sensitivity analysis results assessing the robustness of the statin and cancer association. 21](#_Toc217646901)

## List of Supplementary Figures

[Figure S1. Directed Acyclic Graph for the causal relationship of exposure and outcome 11](#_Toc212027335)

[Figure S2: Fine and gray competing risk model assumption and goodness of fitness. The covariates exhibited a constant effect over time, with minor fluctuations occurring later. 12](#_Toc212027336)

[Figure S3: Propensity score distribution before and after adjustment. The propensity score distribution shows sufficient overlap between the groups after adjustment, indicating that the groups are comparable and support using IPW. 14](#_Toc212027337)

[Figure S4: Standardized mean difference for pair-wise comparisons of characteristics after weighting (lipophilic vs hydrophilic; vs no statin). A weighted value below the dotted line or 0.1 indicates good balance between the groups. 15](#_Toc212027338)

[Figure S5: E values assessing the robustness of the statin and cancer association to unmeasured confounding. 21](#_Toc212027339)

# Graphical Abstract


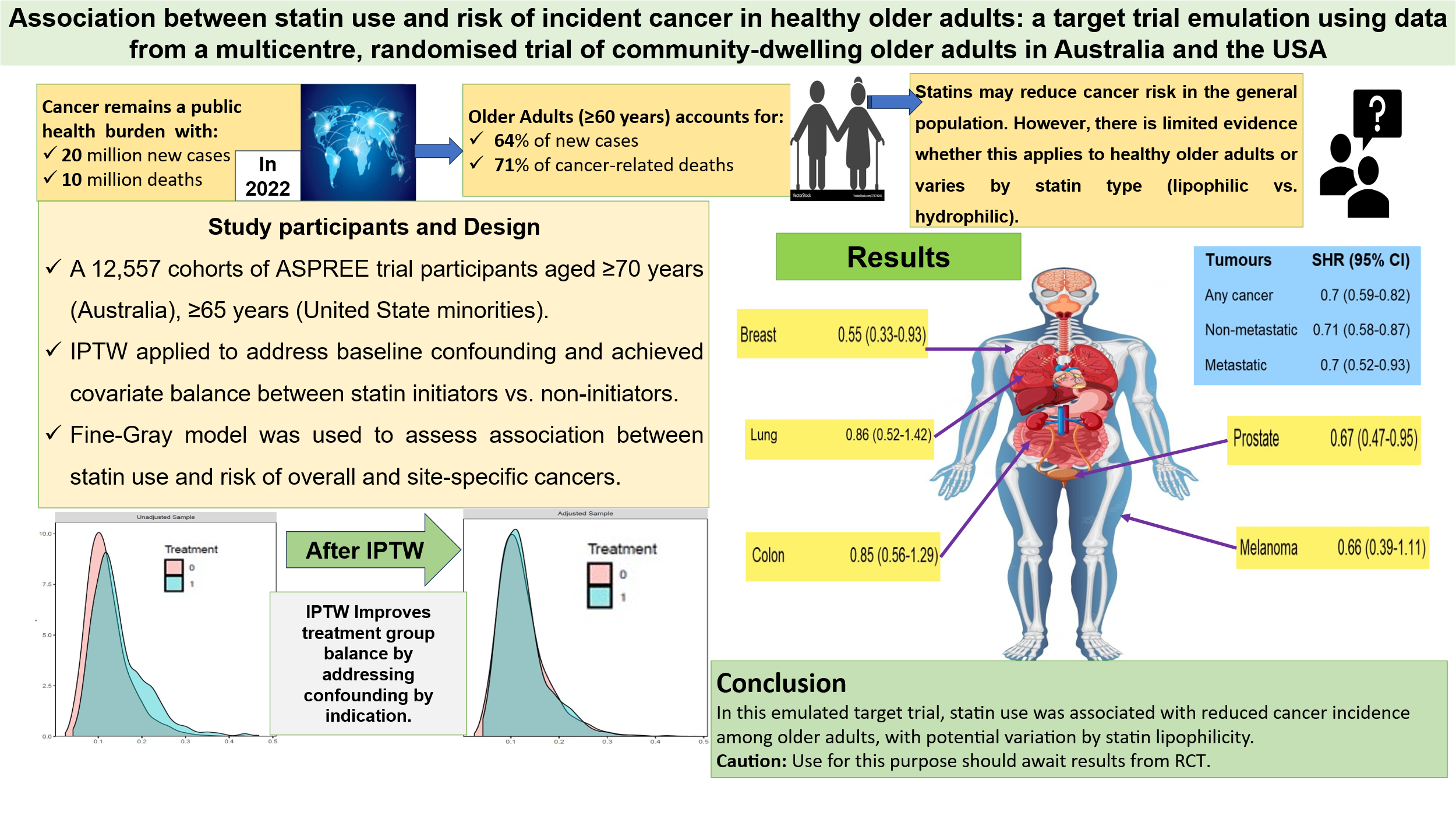


# Supplementary Methods

## Protocol of the hypothetical target trial emulation

Table S1: Specification (protocol) of the target trial and its emulation based on TARGET guideline

| **Criteria** | **Target trial specification** | **Target trial emulation** |
| --- | --- | --- |
| Eligibility criteria | Included healthy older adults aged 70 years or older in Australia (65 or older for USA minorities) and free from dementia cardiovascular disease (CVD), and physical disability.  **Exclusion criteria:**   - History of CVD, dementia, or a Modified Mini-Mental State Examination (3MS) score of less than 77, anaemia, and clinical atrial fibrillation. - Advanced conditions that could cause death in the next five years - Currently use of another anticoagulant or antiplatelet - Systolic blood pressure (SBP) >/= 180 or Diastolic blood pressure (DBP) >/=105, | The Same |
| Treatment strategies | Intervention: Statin use  Control: Placebo | Intervention: Statin use.  Control: Statin non-use. |
| Assignment procedure | Randomization to statin or placebo. | Randomization was replicated using a confounding adjustment method (IPTW). |
| Follow up | From the day of randomization to withdrawal, loss to follow-up, outcome, and at the end of the study (January 2022). | From time zero (one year after randomization date of ASpirin in Reducing Events in the Elderly (ASPREE) study) to withdrawal, loss to follow-up, outcome, and at the end of the study (January 2022). |
| Outcome | Overall and type-specific cancer | Overall and type-specific cancer |
| Causal contrasts | Intention-to-treat effect estimated as a hazard ratio using the Cox proportional hazards model | Intention-to-treat effect estimated as a sub-distribution hazard ratio using the Fine and Gray competing risks model |
| Identifying assumptions (All assumptions were checked through sensitivity analyses or standardized mean difference) | N/A | - Conditional exchangeability: The potential outcomes are independent of treatment assignment, given the measured covariates. |
| Data analysis plan | Intention-to-treat (ITT) analysis using Cox PH regression. | Both ITT (time fixed) and time dependent (TD) analysis using a marginal structural competing risk model. In ITT analysis, the time fixed statin initiation was weighted using baseline covariates, while in TD analysis, the cumulative effect of time-varying statin exposure was estimated, accounting for switching, discontinuation, and change of statin to other types over time. This approach handles the immortal time bias in observational studies.   - Pre-specified subgroup analyses based on sociodemographic, and clinical factors were conducted.   **Sensitivity analysis**   - E value to see the effect of unmeasured confounding. - Positive (change in Low Density Lipoprotein Cholesterol (LDL C) as outcome) and negative (withdrawal from ASPREE as outcome) control analysis - Excluding high and very high LDL-C and Total Cholesterol (TC). - To mitigate potential bias arising from immortal time, we conducted a time-matched nested case-control analysis alongside the time-updated analysis employed as the primary analysis. - Non-statin lipid-lowering therapies were excluded to evaluate the specific impact of statins. |

## Cancer Definition

The primary outcome was overall cancer and site-specific cancers, according to the ASPREE cancer endpoint definition and determined by the ASPREE cancer adjudication committee**^[1]^**.

Cancer events were identified through self-reports, review of medical records held at general practice, or linkage with the National Death Index. The ASPREE Endpoint team then coded these events (to remove duplicates or non-genuine triggers, such non-melanoma skin cancers) and gathered supporting documents (e.g., imaging results, specialist letters, histopathology results, hospital progress or discharge notes) from various sources (e.g., general practice, hospital records, specialist records, pathology providers) to create an event summary. Two independent blinded clinical experts used this summary to confirm or refute the report and categorize the case based on ASPREE criteria, with a third adjudicator utilised to resolve any disagreements. Adjudicators confirmed anatomical type, disease extent (non-metastatic vs metastatic), date and stage at diagnosis. This process did not apply to pre-randomization cancers, which were self-reported by participants at enrolment.

Table S2. Cancer diagnosis criteria used in ASPREE

|  | ASPREE Cancer Criteria |
| --- | --- |
| Confirmed cancer endpoint (endpoint = yes) | - Incident non-metastatic cancer diagnosed post-randomization - Incident metastatic cancer diagnosed post-randomization - Metastasis from pre-existing cancer diagnosed post-randomization - Incident blood cancer diagnosed post-randomisation |
| Refuted cancer endpoint (endpoint = no) | - Cancer is not present - Carcinoma in-situ - Case lacks supporting evidence (e.g. histopathology report, imaging report or document of strong clinical evidence) required to make a definitive cancer diagnosis |
| Details for confirmed endpoints | - The incident cancer will comprise every new cancer type diagnosis reported and confirmed following treatment assignment. - Within three months of diagnosis, new cancers that showed no signs of distant metastatic spread were deemed to be localized at presentation. - Within three months of presentation, cancers that showed indications of distant metastatic dissemination or distal nodal involvement were considered metastatic at the time of presentation. - Any non-localized (distant) recurrence of the initial tumour was considered a metastatic recurrence among study participants with a prior cancer diagnosis, whereas incident cancers comprised any new cancer type. |

**Note that** non-melanoma skin cancer was not considered to be cancer endpoints unless they found within the head and neck (lip, tongue, cheek) or they resulted in metastasis or death.

# Missing data

Table S3: Missing baseline covariate data among statin initiators and non-initiators

| Covariates with missing data | Non-initiators (N=10961) | Statin initiators (N=1596) | Total (N=12557) |
| --- | --- | --- | --- |
| Body Mass Index | 49(0.4%) | 3(0.2%) | 52 (0.4) |
| Metabolic syndrome | 123 (1.2%) | 10(0.6%) | 133 (1.1%) |
| Low Density Lipoprotein Cholesterol | 274 (2.6%) | 37(2.3%) | 311 (2.5%) |
| Ratio of total cholesterol to high density lipoprotein cholesterol | 257(2.3%) | 27(1.7%) | 284 2.3%) |
| Ratio of triglyceride to high density lipoprotein cholesterol | 258 (2.4%) | 28 (1.8%) | 286 2.3%) |
| Waist circumference to height ratio | 145(1.3%) | 19 1.2%) | 164 (1.3%) |
| Estimated glomerular filtration rate | 275(2.5%) | 39 (2.4%) | 314 2.5%) |

## Study’s Power and detectable effect size

We determined the detectable effect size assuming an 80% power using Freedman's approach for power calculation**^[2, 3]^**. The effect size analysis indicates that with 80% power, our sample can detect a minimum of 18%, 25%, and 30% reduction in the hazard ratio (HR) for any cancer, non-metastatic cancer, and metastatic cancer, respectively (Table S2). This indicates that our sample size is adequate to identify the observed effects with 80% power, as all the detectable effect sizes are smaller than the observed effect size.

Table S4: Detectable effect size at 80% of the study and observed effect size.

| **Cancers** | **Detectable effect size (reduction in %) at 80% of power** | **Observed effect size (reduction %)** |
| --- | --- | --- |
|  |  |  |
| Any cancer | 18 | 30 |
| Non metastatic | 25 | 29 |
| Metastatic | 30 | 30 |

Table S5: Definition of covariates.

| Confounder | Definition |
| --- | --- |
| Hypertension | Mean of three blood pressure readings with SBP ≥ 140 mmHg, DBP ≥ 90 mmHg, or use of antihypertensive medication. |
| Diabetes mellitus | Fasting blood glucose (FBG) ≥ 126 mg/dL based on American Diabetes Association (ADA) **^[4]^** and WHO criteria**^[5]^**, diabetes treatment, or self-reported diabetes mellitus. |
| Metabolic syndrome | ATP III criteria for diagnosing metabolic syndrome**^[6]^**. Accordingly, metabolic syndrome is defined by the presence of any three of the following five criteria: abdominal obesity, characterized by a waist circumference of levels below 102 cm in men or 88 cm in women; elevated triglycerides, with levels of 150 mg/dL or higher, or undergoing treatment for elevated triglycerides; reduced High Density Lipoprotein Cholesterol (HDL C), with levels below 40 mg/dL in men or 50 mg/dL in women, or undergoing treatment for low HDL; elevated blood pressure, with readings of 130/85 mmHg or higher, or undergoing treatment for hypertension; and elevated fasting glucose, with levels of 100 mg/dL or higher, or undergoing treatment for diabetes mellitus. |
| Chronic Kidney Disease | Self-reported or eGFR < 60 ml/min/1.73 m²**^[7]^**. |
| Pulmonary disease/respiratory condition | Self-reported asthma or use of medication for obstructive pulmonary disorder. |
| Parkinson’s Disease | Self-reported or use of antiparkinsonian medication. |
| Personal cancer history | Self-report |
| Gout | Self-report or use of gout medication. |
| Frailty | Defined based on Fried phenotype and deficit accumulation Frailty Index**^[8]^**. |
| Statin and other concomitant medication use | The main exposure and concomitant medications were based on self-report, with participants asked to provide their prescribed medications or a list. Trade and/or generic names were recorded and classified using the WHO’s Anatomical Therapeutic Chemical (ATC) Classification System (<https://www.whocc.no/atc_ddd_index/>). |

## Inverse Probability Weighting

In non-randomized studies, comparisons between exposed and unexposed groups are not straightforward. Shared characteristics linked to both exposure and outcome can confound the relationship, leading to biased true effects. Various statistical techniques are utilized in observational studies to mitigate confounding and evaluate causal relationships, including newer approaches based on propensity scores, such as inverse probability treatment weighting (IPTW). The IPTW method involves weighting by the inverse probability of receiving the treatment (1/(propensity score) for the treated group and 1/ (1−propensity score) for the untreated group)**^[9]^**. Individuals with lower probabilities of exposure who are exposed, and those with higher probabilities of exposure who are unexposed, receive larger weights, thereby increasing their influence on the comparison. Including these weights in the analysis makes assignment to the treatment group independent of the variables in the model. This approach was designed to effectively address group imbalances, thereby yielding more reliable and unbiased estimates of the association between statin use and cancer risk.

## Confounder selection

The confounder or covariates used for propensity score calculation was guided by expert knowledge and previous study **^[10]^**. Accordingly, sociodemographic criteria including age at randomization, residence, sex, race or ethnicity, level of education, alcohol consumption, and smoking status; clinical and laboratory results including SBP, DBP, estimated glomerular filtration are, LDL-C, body mass index (BMI), waist-to-height ratio, TC to HDL-C ratio, and triglyceride to HDL-C ratio; a family history of CVD and cancer; a personal history of cancer; participant’s CVD risk score (using PREDICT-1 equation)**^[11]^**; comorbidities and chronic conditions including diabetes mellitus, depression, Chronic Kidney Disease, hypertension, gout, pulmonary disease, and metabolic syndrome; and relevant medications such as metformin, nonsteroidal anti-inflammatory agents, antidepressants, antihypertensive (Angiotensin converting enzyme inhibitors, calcium channel blocker, angiotensin receptor blockers, beta-blockers, and diuretics), non-statin lipid lowering medications and antithrombotic use were all selected to weight the treatment.

## Directed Acyclic Graph

We used a Directed Acyclic Graph (DAG) to show the relationship between the included covariates, statin use and cancer outcome. The DAG was constructed using the DAGitty web ([DAGitty v3.1](https://dagitty.net/dags.html)) application **^[12]^**. In this graph, pink bubbles represent the covariates, green bubbles denote the exposures, and the blue bubble shows the outcome. Pink lines illustrate biasing pathways, while green lines depict causal pathways.


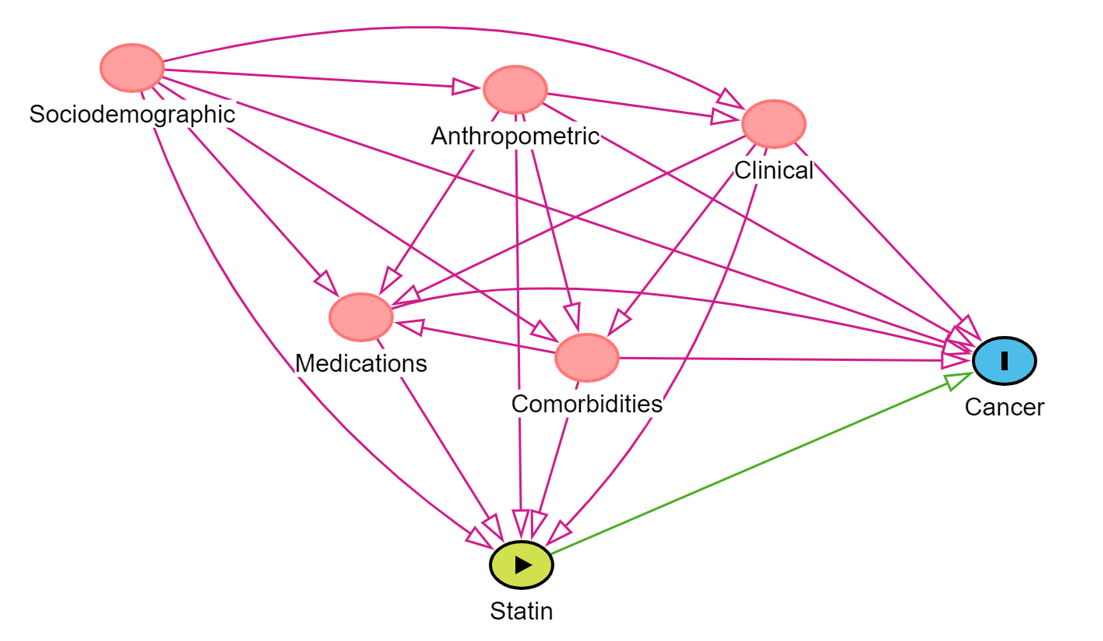


Figure S1. Directed Acyclic Graph for the causal relationship of exposure and outcome

## Proportional Hazard Assumption

The Fine and Gray model is frequently utilized for analysing competing risks data, with the assumption that all covariates exert constant effects over time**^[13]^**. To test the validity of this assumption, we plotted coefficients that allowed covariates to vary over time to inspect if any specific covariate demonstrated a time-varying effect visually**^[14]^**. Additionally, we evaluated the Proportional Hazards Assumption (PHA) using a log-log plot, as shown below.


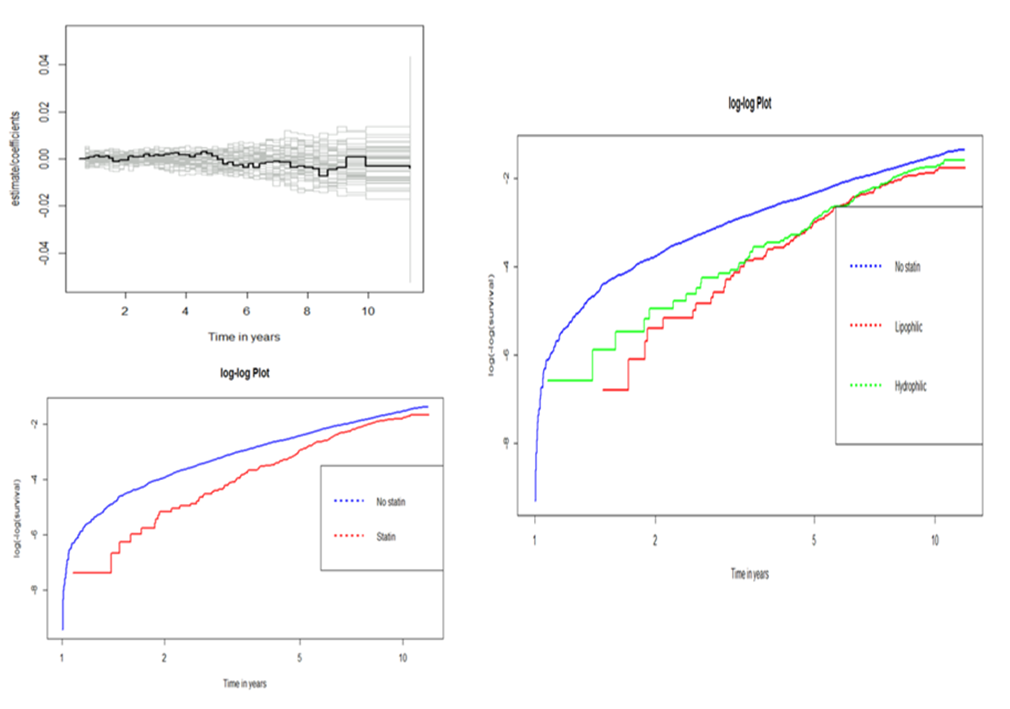


Figure S2: Fine and gray competing risk model assumption and goodness of fitness. The covariates exhibited a constant effect over time, with minor fluctuations occurring later.

# Supplementary Results

## Distribution of individual statin medications

During the study period, most participants were taking atorvastatin, followed by rosuvastatin and simvastatin (Table S4).

Table S6: Distribution of individual statin medications

| Sample | Total statin initiators | Lipophilic | Hydrophilic |
| --- | --- | --- | --- |
| 12557 | 1596 | 882 | 714 |
| **Individual medications** | | | |
| Atorvastatin |  | 698 |  |
| Fluvastatin |  | 1 |  |
| Lovastatin |  | 3 |  |
| Simvastatin |  | 180 |  |
| Pravastatin |  |  | 88 |
| Rosuvastatin |  |  | 626 |

## Weight distribution and covariate balance plot

We evaluated the covariate balance between the groups by examining the standardized mean differences attributed to the propensity score model, both before and after weighting**^[15]^**. A value of less than 0.1 after adjustment (below the dashed reference line) indicated good balance. Figures S4a and S4b show that, for both the primary and secondary analyses, no value exceeded 0.1, indicating that the groups had similar characteristics given the covariates.

Table S7: Distribution of inverse probability treatment weights

|  | **Mean (SD)** | **Median (IQR)** | **5%** | **95 %** | **Mini** | **Max** |
| --- | --- | --- | --- | --- | --- | --- |
| For target trial emulation of Statin vs, no statin (two arms) | | | | | | |
| IPTW | 1.00 (0.16) | 0.98 (0.95, 1.03) | 0.85 | 1.21 | 0.20 | 3.15 |
| For target trial emulation of statin types (three arms) | | | | | | |
| IPTW | 1.00(0.18) | 0.98(0.95, 1.03) | 0.83 | 1.21 | 0.17 | 3.99 |


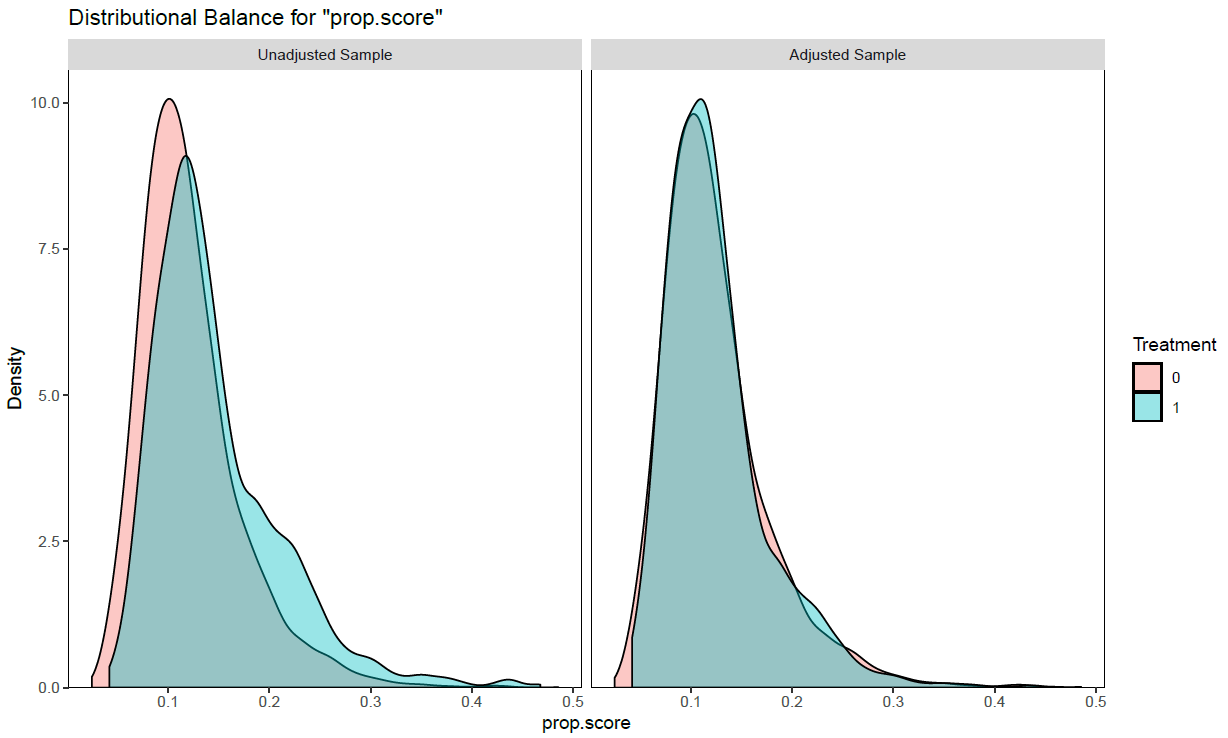


Figure S3: Propensity score distribution before and after adjustment. The propensity score distribution shows sufficient overlap between the groups after adjustment, indicating that the groups are comparable and support using IPW.


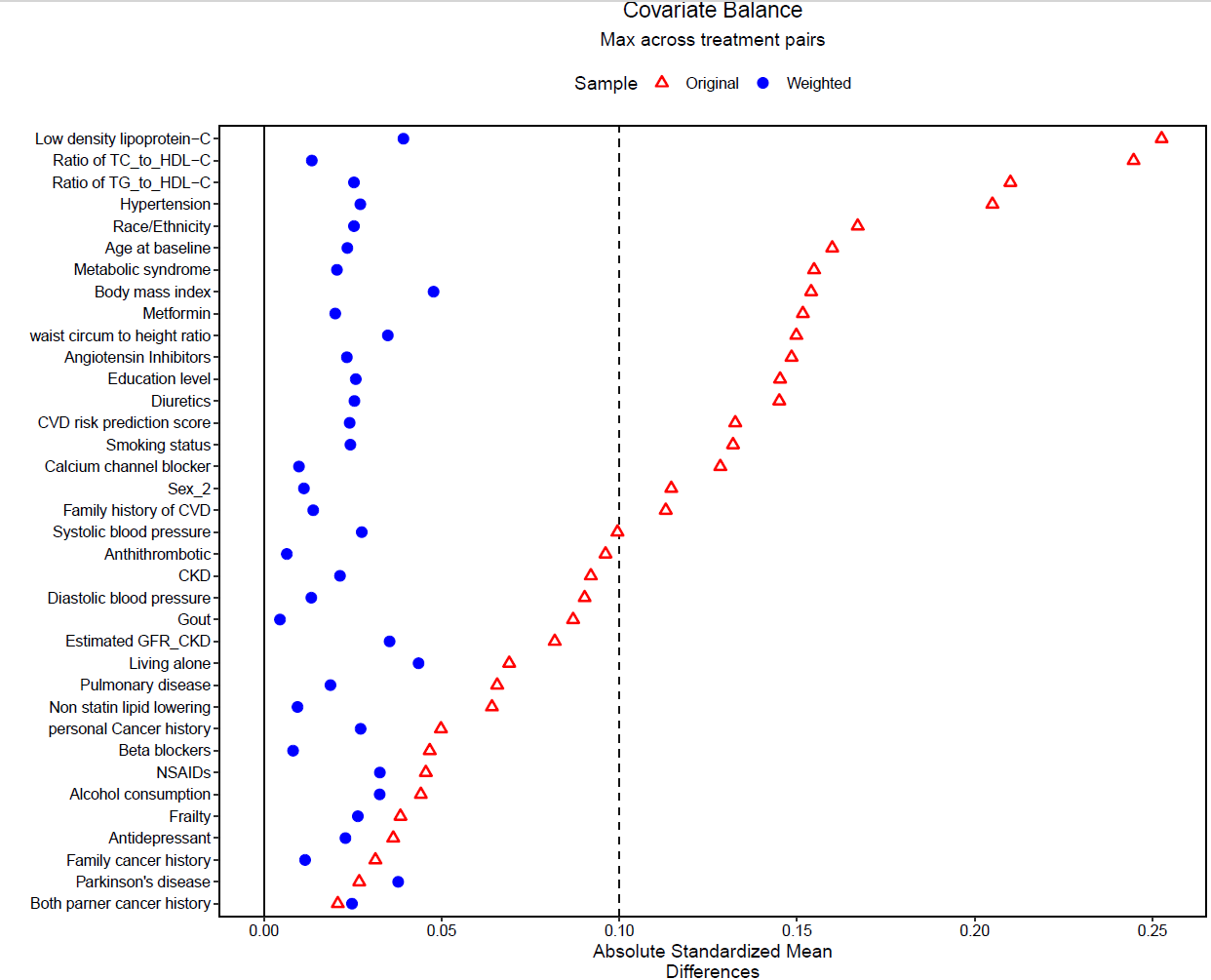


Figure S4: Standardized mean difference for pair-wise comparisons of characteristics after weighting (lipophilic vs hydrophilic; vs no statin). A weighted value below the dotted line or 0.1 indicates good balance between the groups.

## Secondary analysis (lipophilic vs hydrophilic; vs no statin)

Table S8: Demographic and health characteristics before and after weighting for statin types (lipophilic vs hydrophilic) vs. no statin.

|  | **Before weighting** | | | | **After weighting** | | | |
| --- | --- | --- | --- | --- | --- | --- | --- | --- |
|  | Hydrophilic statin (N=714) | Lipophilic statin (N=882) | Non- initiators (N=10961) | Total (N=12557) | Hydrophilic statin (N=716) | Lipophilic statin (N=871) | Non- initiators (N=10962) | Total (N=12548) |
| Age in mean (SD) and n (%) | | | | |  |  |  |  |
| Mean (SD) | 74.5 (4.2) | 75.0 (4.7) | 75.2 (4.6) | 75.2 (4.6) | 75.1 (4.6) | 75.2 (4.7) | 75.2 (4.6) | 75.2 (4.6) |
| 65-69 | 14 (2.0) | 52 (5.9) | 281 (2.6) | 347 (2.8) | 23 (3.2) | 23 (2.7) | 304 (2.8) | 350 (2.8) |
| 70-74 | 448 (62.7) | 450 (51.0) | 6088 (55.5) | 6986 (55.6) | 385 (53.9) | 479 (55.0) | 6094 (55.6) | 6959 (55.5) |
| 75-79 | 170 (23.8) | 245 (27.8) | 2831 (25.8) | 3246 (25.9) | 196 (27.4) | 229 (26.3) | 2836 (25.9) | 3261 (26.0) |
| 80-84 | 61 (8.5) | 112 (12.7) | 1289 (11.8) | 1462 (11.6) | 81 (11.3) | 107 (12.2) | 1277 (11.6) | 1464 (11.7) |
| >=85 | 21 (2.9) | 23 (2.6) | 472 (4.3) | 516 (4.1) | 31 (4.3) | 33 (3.8) | 450 (4.1) | 514 (4.1) |
| Gender (female)-n (%) | 353 (49.4) | 447 (50.7) | 6087 (55.5) | 6887 (54.8) | 388 (54.3) | 474 (54.5) | 6019 (54.9) | 6882 (54.8) |
| Education n (%) | | | |  |  |  |  |  |
| 12-15 | 198 (27.7) | 288 (32.7) | 3165 (28.9) | 3651 (29.1) | 199 (27.8) | 261 (30.0) | 3185 (29.1) | 3645 (29.0) |
| 15+ | 165 (23.1) | 224 (25.4) | 3033 (27.7) | 3422 (27.3) | 194 (27.1) | 230 (26.3) | 2990 (27.3) | 3413 (27.2) |
| 9-11 | 351 (49.2) | 370 (42.0) | 4763 (43.5) | 5484 (43.7) | 323 (45.1) | 380 (43.7) | 4787 (43.7) | 5490 (43.8) |
| Race/Ethnicity n (%) | | | |  |  |  |  |  |
| Black American | 24 (3.4) | 83 (9.4) | 443 (4.0) | 550 (4.4) | 35 (4.9) | 39 (4.4) | 483 (4.4) | 557 (4.4) |
| Latino | 18 (2.5) | 26 (2.9) | 231 (2.1) | 275 (2.2) | 17 (2.4) | 21 (2.4) | 240 (2.2) | 278 (2.2) |
| White | 662 (92.7) | 759 (86.1) | 10161 (92.7) | 11582 (92.2) | 657 (91.8) | 801 (91.9) | 10108 (92.2) | 11566 (92.2) |
| Others | 10 (1.4) | 14 (1.6) | 126 (1.1) | 150 (1.2) | 6 (0.9) | 11 (1.3) | 130 (1.2) | 148 (1.2) |
| Smoking | | | |  |  |  |  |  |
| Current | 37 (5.2) | 42 (4.8) | 386 (3.5) | 465 (3.7) | 23 (3.2) | 29 (3.3) | 404 (3.7) | 455 (3.6) |
| Former | 317 (44.4) | 382 (43.3) | 4363 (39.8) | 5062 (40.3) | 287 (40.0) | 349 (40.0) | 4415 (40.3) | 5051 (40.2) |
| Never | 360 (50.4) | 458 (51.9) | 6212 (56.7) | 7030 (56.0) | 406 (56.8) | 493 (56.6) | 6142 (56.0) | 7042 (56.1) |
| Living alone- n (%) | 209 (29.3) | 285 (32.3) | 3565 (32.5) | 4059 (32.3) | 220 (30.7) | 287 (32.9) | 3546 (32.3) | 4052 (32.3) |
| Alcohol consumption n (%) | | | |  |  |  |  |  |
| Current | 559 (78.3) | 671 (76.1) | 8500 (77.5) | 9730 (77.5) | 546 (76.3) | 670 (76.9) | 8491 (77.5) | 9707 (77.4) |
| Former | 47 (6.6) | 68 (7.7) | 600 (5.5) | 715 (5.7) | 43 (5.9) | 45 (5.2) | 623 (5.7) | 711 (5.7) |
| Never | 108 (15.1) | 143 (16.2) | 1861 (17.0) | 2112 (16.8) | 127 (17.7) | 156 (17.9) | 1847 (16.9) | 2130 (17.0) |
| Family history of CVD- n (%) | 437 (61.2) | 566 (64.2) | 6433 (58.7) | 7436 (59.2) | 420 (58.6) | 522 (59.9) | 6491 (59.2) | 7433 (59.2) |
| Family history of cancer- n (%) | 428 (59.9) | 520 (59.0) | 6634 (60.5) | 7582 (60.4) | 434 (60.7) | 529 (60.8) | 6620 (60.4) | 7584 (60.4) |
| Personal cancer history- n (%) | 124 (17.4) | 163 (18.5) | 2113 (19.3) | 2400 (19.1) | 146 (20.5) | 172 (19.8) | 2095 (19.1) | 2414 (19.2) |
| BMI categories (in kg/m2) n (%) | | | |  |  |  |  |  |
| <25 | 174 (24.4) | 218 (24.7) | 3312 (30.2) | 3704 (29.5) | 218 (30.5) | 256 (29.3) | 3238 (29.5) | 3712 (29.6) |
| 25-30 | 315 (44.1) | 389 (44.1) | 4829 (44.1) | 5533 (44.1) | 316 (44.2) | 376 (43.2) | 4828 (44.0) | 5520 (44.0) |
| >=30 | 225 (31.5) | 275 (31.2) | 2820 (25.7) | 3320 (26.4) | 181 (25.3) | 240 (27.5) | 2896 (26.4) | 3316 (26.4) |
| LDL level categories n (%) | | | | | |  |  |  |
| >100 | 654 (91.6) | 767 (87.0) | 9126 (83.3) | 10547 (84.0) | 602 (84.2) | 743 (85.3) | 9206 | 10551 (84.1) |
| <100 | 60 (8.4) | 115 (13.0) | 1835 (16.7) | 2010 (16.0) | 113 (15.8) | 128 (14.7) | 1756 (16.0) | 1997 (15.9) |
| Comorbidities n (%) | | | | |  |  |  |  |
| Hypertension | 541 (75.8) | 693 (78.6) | 7626 (69.6) | 8860 (70.6) | 512 (71.5) | 625 (71.7) | 7733 (70.5) | 8870 (70.7) |
| Diabetes mellitus | 92 (12.9) | 90 (10.2) | 595 (5.4) | 777 (6.2) | 77 (10.8) | 73 (8.4) | 635 (5.8) | 786 (6.3) |
| Metabolic syndrome | 199 (27.9) | 239 (27.1) | 2314 (21.1) | 2752 (21.9) | 156 (21.8) | 198 (22.8) | 2400 (21.9) | 2754 (21.9) |
| CKD | 130 (18.2) | 171 (19.4) | 1725 (15.7) | 2026 (16.1) | 120 (16.8) | 148 (16.9) | 1767 (16.1) | 2035 (16.2) |
| Gout | 38 (5.3) | 37 (4.2) | 385 (3.5) | 460 (3.7) | 26 (3.6) | 32 (3.6) | 401 (3.7) | 459 (3.7) |
| Pulmonary disease | 105 (14.7) | 110 (12.5) | 1431 (13.1) | 1646 (13.1) | 91 (12.8) | 113 (13.0) | 1438 (13.1) | 1643 (13.1) |
| Frailty n (%) | | | |  |  |  |  |  |
| Pre frail | 275 (38.5) | 341 (38.7) | 4195 (38.3) | 4811 (38.3) | 283 (39.6) | 323 (37.1) | 4211 (38.4) | 4818 (38.4) |
| Frail | 11 (1.5) | 22 (2.5) | 235 (2.1) | 268 (2.1) | 9 (1.3) | 20 (2.3) | 236 (2.2) | 265 (2.1) |
| Parkinson’s disease | 11 (1.5) | 13 (1.5) | 135 (1.2) | 159 (1.3) | 6 (0.9) | 12 (1.4) | 139 (1.3) | 157 (1.3) |
| GORD | 208 (29.1) | 225 (25.5) | 2757 (25.2) | 3190 (25.4) | 196 (27.5) | 217 (24.9) | 2773 (25.3) | 3187 (25.4) |
| CVD risk prediction score n (%) | | | | |  |  |  |  |
|  |  |  |  |  |  |  |  |  |
| Low risk | 465 (65.1) | 527 (59.8) | 7366 (67.2) | 8358 (66.6) | 473 (66.1) | 569 (65.3) | 7299 (66.6) | 8341 (66.5) |
| Moderate risk | 203 (28.4) | 286 (32.4) | 2857 (26.1) | 3346 (26.6) | 188 (26.2) | 238 (27.4) | 2917 (26.6) | 3343 (26.6) |
| High risk | 46 (6.4) | 69 (7.8) | 738 (6.7) | 853 (6.8) | 55 (7.7) | 63 (7.3) | 745 (6.8) | 864 (6.9) |
| Continuous clinical factors – Mean (SD) | | | | | |  |  |  |
| Ratio of TC to HDL level | 4.0 (1.1) | 3.9 (1.1) | 3.6 (1.2) | 3.7 (1.2) | 3.8 (1.1) | 3.8 (1.1) | 3.7 (1.2) | 3.7 (1.2) |
| Ratio of TG to HDL level | 2.5 (2.0) | 2.4 (2.0) | 2.1 (1.7) | 2.1 (1.8) | 2.2 (1.7) | 2.2 (1.8) | 2.1 (1.8) | 2.1 (1.8) |
| Ratio of AC to height | 0.6 (0.1) | 0.6 (0.1) | 0.6 (0.1) | 0.6 (0.1) | 0.6 (0.1) | 0.6 (0.1) | 0.6 (0.1) | 0.6 (0.1) |
| SBP mmHg | 140.2 (16.3) | 141.5 (17.0) | 139.0 (16.6) | 139.2 (16.7) | 139.6 (16.5) | 140.4 (17.0) | 139.2 (16.6) | 139.3 (16.6) |
| DBP mmHg | 78.1 (10.0) | 78.4 (9.9) | 77.4 (10.0) | 77.5 (10.0) | 77.8 (10.0) | 77.7 (9.8) | 77.5 (10.0) | 77.5 (10.0) |
| Estimated GFR | 72.4 (13.7) | 72.6 (14.7) | 73.6 (13.5) | 73.4 (13.6) | 73.0 (13.7) | 73.4 (14.2) | 73.4 (13.6) | 73.4 (13.7) |
| Medications- n (%) | | | | |  |  |  |  |
| OLLM | 37 (5.2) | 51 (5.8) | 481 (4.4) | 569 (4.5) | 37 (5.2) | 50 (5.7) | 485 (4.4) | 572 (4.6) |
| Antithrombotic | 39 (5.5) | 69 (7.8) | 665 (6.1) | 773 (6.2) | 44 (6.2) | 53 (6.1) | 673 (6.1) | 771 (6.1) |
| Metformin | 35 (4.9) | 38 (4.3) | 222 (2.0) | 295 (2.3) | 14 (2.0) | 22 (2.6) | 258 (2.4) | 295 (2.3) |
| NSAIDs | 118 (16.5) | 161 (18.3) | 1916 (17.5) | 2195 (17.5) | 118 (16.5) | 153 (17.6) | 1915 (17.5) | 2186 (17.4) |
| Diuretics | 143 (20.0) | 212 (24.0) | 1987 (18.1) | 2342 (18.7) | 135 (18.8) | 173 (19.9) | 2046 (18.7) | 2354 (18.8) |
| Angiotensin inhibitors | 205 (28.7) | 223 (25.3) | 2429 (22.2) | 2857 (22.8) | 171 (23.9) | 204 (23.5) | 2493 (22.7) | 2869 (22.9) |
| CCB | 139 (19.5) | 159 (18.0) | 1600 (14.6) | 1898 (15.1) | 107 (15.0) | 134 (15.4) | 1657 (15.1) | 1898 (15.1) |
| Beta blockers | 53 (7.4) | 72 (8.2) | 760 (6.9) | 885 (7.0) | 54 (7.6) | 70 (8.0) | 774 (7.1) | 898 (7.2) |
| Antidepressant | 85 (11.9) | 101 (11.5) | 1185 (10.8) | 1371 (10.9) | 75 (10.4) | 98 (11.2) | 1199 (10.9) | 1372 (10.9) |

The 5-year predicted risk is calculated using following formula: ***Predict1 equation*** = (1 − S0^exp (Coeff _age_ * I (age-56.13665) + Coeff _Race_ * Race (Chinese or other Asian) + Coeff _IRSAD_ * I (IRSAD quintile-2.990826) + Coeff _Ex/current smoker_ * Smoking + Coeff _Fam-His-CVD_ * Fam-His-CVD + Coeff _Diabetes_ * Diabetes + Coeff _SBP_ * I (SBP-129.0173) + Coeff _TC/HDL-C_ * I (TC/HDL ratio-3.726268) + Coeff _OBPLM_ * OBPLM + Coeff _OLLM_ * OLLM + Coeff _OATM_ * OATM + Coeff _age * diabetes_ * (I (age-56.13665) * diabetes) + Coeff _age * SBP_ * (I (age-56.13665) * I (SBP-129.0173)) + Coeff _SBP * OBPLM_ * (I (SBP-129.0173)* OBPLM))) * 100. BMI: Body mass index; CCB: Calcium channel blockers, CVD: Cardiovascular disease; HDL-C: High-density lipoprotein cholesterol; LDL-C: Low-density lipoprotein cholesterol; NSAIDs: Non-steroidal anti-inflammatory drugs; TG: Triglyceride; TC: Total cholesterol, Fam-his-CVD: Family history of CVD; OATM: On antithrombotic medication; OBPLM: On blood pressure-lowering medication; OLLM: Other lipid-lowering medication; S0: Baseline survival function (at 5 years); SBP: Systolic blood pressure; TC: Total cholesterol.

## Sensitivity analysis

To ensure the robustness of our findings, we conducted several sensitivity analyses. We performed positive and negative control analyses to assess the robustness of our results to potential biases. A positive control can help validate the study's design and analytical procedures can identify the expected effect. Negative control aids in the identification of potential biases or confounding variables that may falsely show an effect in our primary study. For the positive control, we used LDL-C levels, as they are known to be influenced by statins. For the negative control, we used withdrawal from APREE study, helping to validate our findings.

E-values was calculated to assess the robustness of our findings to unmeasured confounding, following the method by VanderWeele and Ding**^[16]^**. This sensitivity analysis helps determine the minimum strength of association that an unmeasured confounder would need with both the treatment and the outcome to fully explain away the observed treatment association. Additionally, we excluded participants with high total cholesterol and LDL-C levels to ensure the robustness of our comparator groups, as these individuals might have different characteristics.

Finally, we assessed the association of incident cancer and statin after excluding non-statin lipid-lowering treatments given these medications may hinder a clear understanding of the direct effects of statins. This analysis helps to clarify whether the cancer-protective effects of statins are specific to their mechanism of action or related to lipid lowering in general. These independent sensitivity analyses collectively strengthen the validity of our findings by addressing various potential sources of bias and confounding.


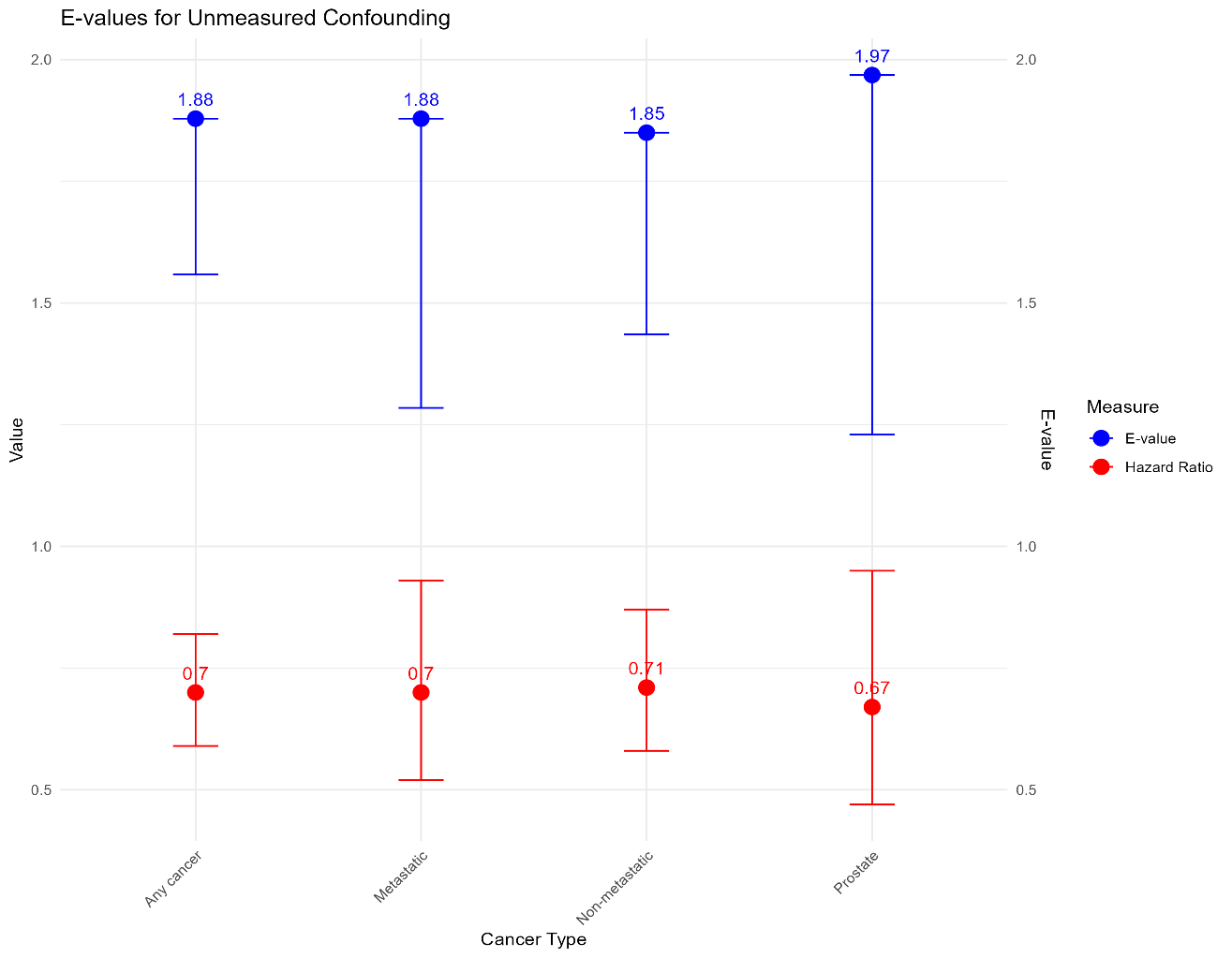


Figure S5: E values assessing the robustness of the statin and cancer association to unmeasured confounding.

Table S9: Sensitivity analysis results assessing the robustness of the statin and cancer association.

| Outcome | HR/Coef. (95% CI) |
| --- | --- |
| **Sensitivity analysis** | |
| Positive and negative control | |
| LDL C in mg/dL (positive control) | -30.3 (-31.2, -29.5) |
| Withdrawal (negative control) | 1.00 (0.79, 1.26) |
| Excluding those with high lipid profiles at baseline. | |
| Excluding total cholesterol >/=290 mg/dL | 0.70 (0.60, 0.82) |
| Excluding LDL-C >/=160 mg/dL | 0.72 (0.60, 0.86) |
| Other Sensitivity analysis | |
| Time matched nested case control **^a^** | 0.77 (0.67, 0.88) |
| Excluding non-statin lipid lowering medication | 0.70 (0.60, 0.83) |

**^a^** To address immortal time bias, we employed a time-matched nested case-control design in addition to the time dependent analysis. Each case was matched to one control based on time since ASPREE randomization, ensuring that the control was at risk at the time the case occurred. The matching ratio was 1:1. After matching, we applied inverse probability of treatment weighting (IPTW) to adjust for all covariates included in the main analysis, thereby preserving comparability between groups beyond the time dimension. We then fitted a conditional logistic regression model using the clogit function from the survival package in R, which accounts for the matched design and allows for estimation of the association between exposure and outcome while controlling for confounding. This approach ensures that the temporal alignment between cases and controls is maintained, minimizing bias due to differential follow-up time.

# Reference

1. McNeil JJ, Gibbs, * Peter, Orchard SG, Lockery JE, Bernstein WB, Cao Y, et al. Effect of aspirin on cancer incidence and mortality in older adults. JNCI: Journal of the National Cancer Institute. 2021;113(3):258–65.

2. Freedman LS. Tables of the number of patients required in clinical trials using the logrank test. Statistics in medicine. 1982;1(2):121–9.

3. Rosner B. Fundamentals of biostatistics: Thomson-Brooks. Cole,(175-176). 2006.

4. Association AD. 2. Classification and diagnosis of diabetes: standards of medical care in diabetes—2021. Diabetes care. 2021;44(Supplement_1):S15–S33.

5. Kononenko IV, Smirnova OM, Mayorov AY, Shestakova MV. Classification of diabetes. World Health Organization 2019. What’s new? Diabetes mellitus. 2020;23(4):329–39.

6. Eckel RH, Grundy SM, Zimmet PZ. The metabolic syndrome. The lancet. 2005;365(9468):1415–28.

7. Levey AS, Coresh J. Chronic kidney disease. The lancet. 2012;379(9811):165–80.

8. Espinoza S, Ernst M, Ryan J, Beilin L, Stocks N, Ward S, et al. The Association between Metabolic Syndrome, Frailty and disability-free survival in Healthy Community-dwelling older adults. The Journal of nutrition, health and aging. 2023;27(1):1–9.

9. Desai RJ, Franklin JM. Alternative approaches for confounding adjustment in observational studies using weighting based on the propensity score: a primer for practitioners. bmj. 2019;367.

10. Jeong GH, Lee KH, Kim JY, Eisenhut M, Kronbichler A, van der Vliet HJ, et al. Effect of Statin on Cancer Incidence: An Umbrella Systematic Review and Meta-Analysis. J Clin Med. 2019;8(6).

11. Pylypchuk R, Wells S, Kerr A, Poppe K, Riddell T, Harwood M, et al. Cardiovascular disease risk prediction equations in 400 000 primary care patients in New Zealand: a derivation and validation study. The Lancet. 2018;391(10133):1897–907.

12. Textor J, Van der Zander B, Gilthorpe MS, Liśkiewicz M, Ellison GT. Robust causal inference using directed acyclic graphs: the R package ‘dagitty’. International journal of epidemiology. 2016;45(6):1887–94.

13. Beyersmann J, Latouche A, Buchholz A, Schumacher M. Simulating competing risks data in survival analysis. Statistics in medicine. 2009;28(6):956–71.

14. Scheike TH, Zhang M-J. Analyzing competing risk data using the R timereg package. Journal of statistical software. 2011;38(2).

15. Imai K, Ratkovic M. Covariate balancing propensity score. Journal of the Royal Statistical Society Series B: Statistical Methodology. 2014;76(1):243–63.

16. VanderWeele TJ, Ding P. Sensitivity analysis in observational research: introducing the E-value. Annals of internal medicine. 2017;167(4):268–74.
